# An Integrated Quantitative Proteomics Workflow for Cancer Biomarker Discovery and Validation in Plasma

**DOI:** 10.3389/fonc.2020.543997

**Published:** 2020-09-23

**Authors:** Vipin Kumar, Sandipan Ray, Saicharan Ghantasala, Sanjeeva Srivastava

**Affiliations:** Department of Biosciences and Bioengineering, Indian Institute of Technology Bombay, Mumbai, India

**Keywords:** cancer biomarker, multiplexed quantitative proteomics, targeted proteomics, label-free quantitation, multiple reaction monitoring, parallel reaction monitoring

## Abstract

Blood plasma is one of the most widely used samples for cancer biomarker discovery research as well as clinical investigations for diagnostic and therapeutic purposes. However, the plasma proteome is extremely complex due to its wide dynamic range of protein concentrations and the presence of high-abundance proteins. Here we have described an optimized, integrated quantitative proteomics pipeline combining the label-free and multiplexed-labeling-based (iTRAQ and TMT) plasma proteome profiling methods for biomarker discovery, followed by the targeted approaches for validation of the identified potential marker proteins. In this workflow, the targeted quantitation of proteins is carried out by multiple-reaction monitoring (MRM) and parallel-reaction monitoring (PRM) mass spectrometry. Thus, our approach enables both unbiased screenings of biomarkers and their subsequent selective validation in human plasma. The overall procedure takes only ~2 days to complete, including the time for data acquisition (excluding database searching). This protocol is quick, flexible, and eliminates the need for a separate immunoassay-based validation workflow in blood cancer biomarker investigations. We anticipate that this plasma proteomics workflow will help to accelerate the cancer biomarker discovery program and provide a valuable resource to the cancer research community.

## Introduction

Plasma is an attractive and reliable sample for cancer research due to its easy accessibility, and plasma proteome can provide a plethora of important information regarding the normal physiological states as well as the cancer-induced alterations in our body (1, 2). Importantly, recent studies showed whole blood as a specimen for liquid biopsy for personalized medicine applications and monitoring the therapeutic responses to the treatment of cancers (3, 4). Mass spectrometry (MS)—based label-free and multiplexed label-based proteomics profiling of the plasma or serum proteome is widely used for unbiased discovery of potential biomarkers for diverse types of human diseases including cancers, infectious diseases, cardiovascular and metabolic disorders (2, 5–8).

In recent years, multiple-reaction monitoring (MRM) and parallel-reaction monitoring (PRM) mass spectrometry approaches have emerged as attractive alternatives for protein immunoassays (9). These targeted proteomics approaches can accurately measure concentrations of multiple proteins in complex biological samples, such as plasma (10–13). Importantly, results obtained in multiplexed MRM/PRM-MS assays correlate well with immunoassay-based measurements (10, 14). One key advantage of these targeted MS-assays is that these allow quantification of variants and modified forms of the proteins by targeting their specific peptide sequences (15, 16). Quantification by traditional immunoassay-based techniques such as Western blotting is based on a single antibody that is often inadequately characterized and protein quantification solely depends on a single signal (17). On the contrary, the quality of the isotopically labeled reference peptides used in MRM or PRM-based methods could be easily evaluated by a fragment ion spectrum and these approaches use multiple signals for obtaining more reliable and robust quantification (17). Moreover, immunoassay-based techniques are often difficult to perform for multiple targets due to the low-throughput of these approaches and the unavailability of suitable antibodies for many proteins. To this end, MRM and PRM-based approaches allow the accurate quantification of hundreds of peptides in a single injection/run of mass spectrometry and therefore are more high-throughput compared to the conventional immunoassay-based measurements. Consequently, a combined workflow involving both discovery and validation phase quantitative proteomics techniques would be extremely beneficial for cancer biomarker research.

**Figure 1:**
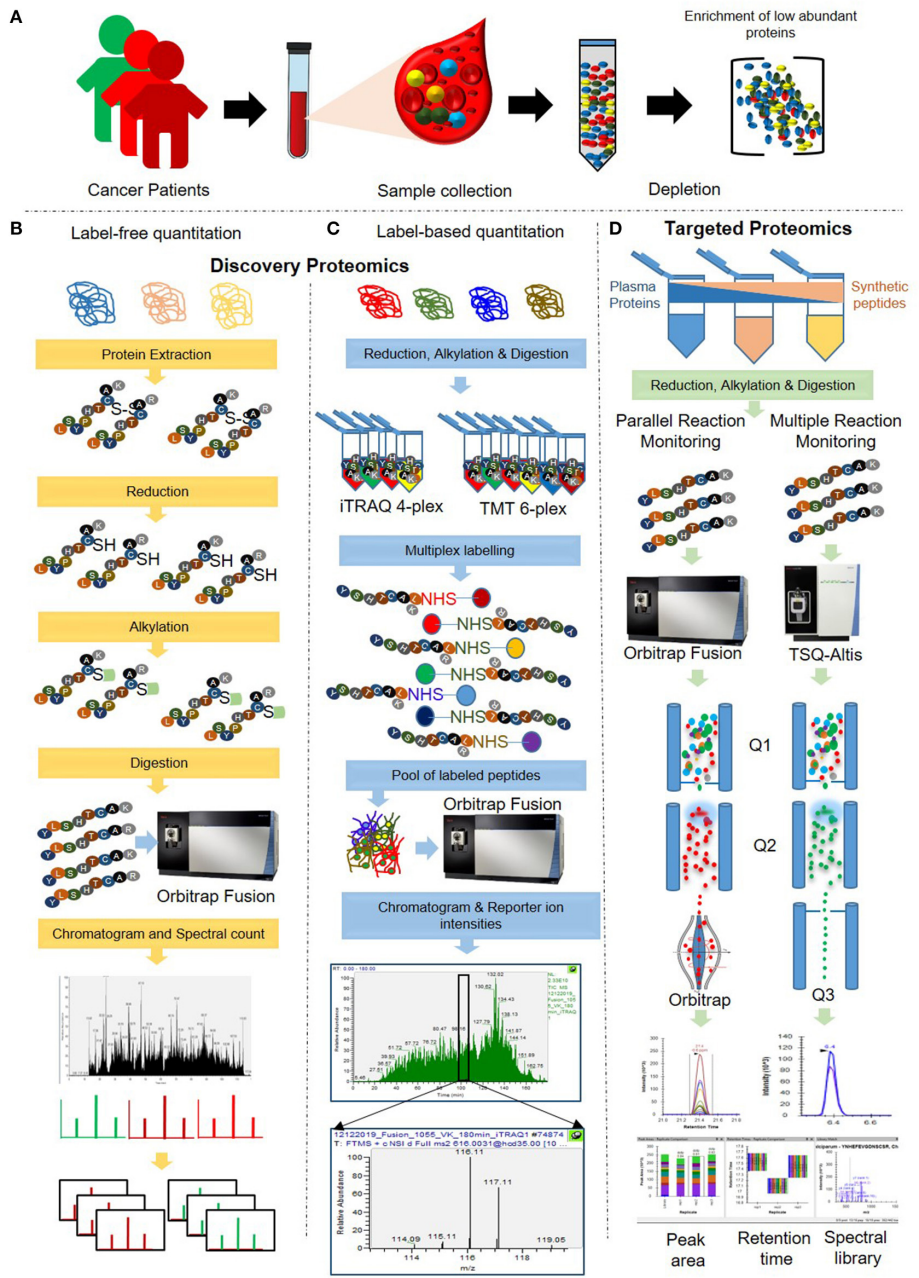
Schematic representation of the integrated workflow for cancer biomarker discovery and validation in plasma. **(A)** Crude plasma samples were depleted using depletion columns for the removal of the top 12 highly abundant proteins. **(B)** Depleted plasma samples were subjected to in-solution digestion, and MS analysis was performed using a label-free quantitation approach. **(C)** The digested peptides were labeled using iTRAQ/TMT reagents and subjected to MS analysis for label-based quantitation. **(D)** Samples were prepared by spiking with the varying amounts of heavy labeled synthetic peptides. Targeted quantification of the spiked synthetic peptides and a few selected potential cancer markers was carried out using Multiple Reaction Monitoring (MRM) and Parallel Reaction Monitoring approach (PRM) approaches.

There are several methods describing sample processing for quantitative proteomics analysis of plasma samples in various cancers, while we have demonstrated here a combined method for both discovery and validation of protein markers in plasma samples. In this respect, we have extensive experience of applying label-based multiplexed quantitative proteomics for the discovery of biomarker panels in cancer and other diseases (18–24). Such multiplexing using stable isotope labeling results in increased throughput, higher precision, better reproducibility, reduced technical variations and fewer missing values (8, 20, 25–30). Further, proteomic profiling using label-free quantitation (LFQ) is another attractive method for cancer biomarker quantification (23, 31). In recent years, we have reported targeted quantitation of proteins by Multiple Reaction Monitoring (MRM) mass spectrometry (18, 32). Here, we have described an amalgamated analysis pipeline for plasma biomarker analysis by integrating the know-how of different quantitative & targeted proteomics methods (Figures 1A–D).

## Experimental Design

In this integrated quantitative proteomics pipeline, three biological pool of plasma samples were analyzed for obtaining a comprehensive proteome profile, and subsequent validation of a few selective peptides. Each of the three plasma pools (named as samples A, B, and C) was a uniform mixture of ten different individual plasma samples. In order to perform targeted proteomics analyses, a pool of 21 heavy labeled synthetic peptides were spiked into the plasma samples at a different ratio. Global quantitative proteomics was performed using both label-free and label-based such as Isobaric tags for relative and absolute quantitation (iTRAQ 4-plex) and Tandem Mass Tag™ (TMT 6-plex) quantitation approaches (Figures 1B,C), while the targeted proteomics was carried out using MRM and PRM-based MS assays (Figure 1D). In iTRAQ experiment, we have used different amount of digested peptides per label to determine the minimum amount of peptides to be labeled and the accuracy of the quantitation.

This protocol consists of label-free and label-based (iTRAQ and TMT) proteome profiling methods for cancer biomarker discovery, followed by the targeted approaches (MRM and PRM) for validation of a few potential marker proteins.

## Stepwise Procedure

### Plasma Sample Preparation Timing 20 min

1. Collect the blood samples into anticoagulant-treated tubes e.g., EDTA-treated or citrate-treated tubes.
  **CRITICAL:** Avoid the use of heparin tubes, heparin can often be contaminated with endotoxin, which can stimulate white blood cells to release cytokines.
2. Remove the cells from blood by centrifugation for 15 min at 2,000 × g, and the resulting supernatant will be plasma.
  **CRITICAL STEP:** The temperature should be maintained at 2–8°C while handling the samples.
  **PAUSE POINT:** The samples can be aliquoted in 0.5 ml tubes and stored in −80°C for long-term storage (6–8 months).

### Depletion of High Abundant Proteins Timing 1 h

3. Equilibrate the depletion spin column at room temperature (room temperature is 25°C).
4. Remove the column screw cap and add 15 μl of crude plasma sample directly to the resin slurry in the column.
  **CRITICAL STEP:** Ensure resin slurry is not dried, and the protein concentration of the plasma sample is around 50–60 μg/μl.
5. Cap the column and invert the column several times until the resin is completely suspended in the solution.
6. Incubate the mixture in the column with gentle end-to-end mixing for 60 min at room temperature. Alternatively, vortex every 5 min.
  **CRITICAL STEP:** Make sure the sample mixes with the resin during the incubation period.
7. Twist off the bottom closure and loosen the cap. Place column into a 2 ml collection tube and centrifuge at 1000 × g for 2 min.
8. Discard the column containing the resin.
9. The filtrate contains depleted plasma (vol. 300 μl approx.) with the top 12 proteins removed.

### Protein Quantification and Sample Preparation for Digestion Timing 2 h

10. Reduce the volume of plasma samples up to 75–100 μl using vacuum centrifuge and quantify using Bradford's reagent following the manufacturer's instruction.
11. Check the quality of depleted plasma samples by running on SDS-PAGE and take 50 μg of proteins and dry it completely.
12. Denature the plasma samples by adding 10 μl of 6 M urea.
13. Reduce disulfide bonds by adding tris (2-carboxyethyl) phosphine (TCEP) to a final concentration of 20 mM. Incubate the sample at 37°C for 60 min.
14. Alkylate reduced cysteine residues by adding iodoacetamide (IAA) to a final concentration of 40 mM. Incubate at room temperature (RT) in the dark for 30 min.

### Enzymatic Digestion of Plasma Proteins Timing 6–8 h

15. Further, dilute the urea concentration by adding 50 mM ammonium bicarbonate in a 1:6 ratios.
  **CRITICAL STEP:** The urea concentration should be <1 M. If you are using trypsin as a digestion enzyme.
16. Add trypsin (Pierce) at an enzyme/substrate ratio of 1:50 and incubate at 37°C with shaking on a table-top shaker set at 500 rpm for 6–8 h.
17. Stop the digestion with formic acid (FA) to a final concentration of 1%.

### Desalting of the Digested Peptides Timing 1 h per 5–10 Samples

18. Prepare the C18 desalting column by using Empore C18 extraction disks. Pack one plug of C18 material into each stage tip (200 μl pipette tips) for a total binding capacity of ~25 μg total. Create extraction disks using 200 μl tips, as shown in Figure S1.
19. Activate the desalting column with 50 μl of methanol. Centrifuge at 1,000 g for 2 min at RT and discard the liquid from the collection vial. Repeat this step two times.
  **CRITICAL:** All subsequent centrifugation steps for desalting are for the same duration at the same speed and RT.
20. Wash the desalting column with 50 μl of acetonitrile. Centrifuge at 1,000 g for 2 min at RT and discard the liquid from the collection vial. Repeat this step two times.
21. Equilibrate the desalting column twice with 50 μl of 0.1% (v/v) FA (solvent A). Repeat the centrifugation step. Discard the liquid.
22. Reconstitute the samples in 50 μl of 0.1% (v/v) FA and vortex for 10 min. at slow speed.
23. Pass the reconstituted samples through the desalting column using either syringe or centrifuge at 1,000 g for 2 min. Repeat this step at least 4 times.
  **CRITICAL:** Ensure that there is no trapping of the air bubble in the desalting column.
24. Store the flow-through at 4°C.
25. Wash the samples twice with 50 μl of solvent A. Repeat the centrifugation step. Discard the liquid.
26. Pass 50 μl of 40% (v/v) acetonitrile in 0.1% (v/v) FA and collect the eluate in new 1.5 ml screw cap. Repeat this step with 50% and 60% (v/v) acetonitrile in 0.1% (v/v) FA and collect the eluate in same vial.
27. Dry 150 μl of desalted sample using a speed vacuum centrifuge.
  **PAUSE POINT:** Store the desalted peptides at−20°C till further process.

### Quantification of the Desalted Peptides Timing 10 min

28. Reconstitute the desalted peptides in 0.1% (v/v) FA.
29. Wipe the μDrop plate with 70% ethanol using lint-free tissue papers.
  **CRITICAL:** Avoid using normal tissue paper and 70% isopropanol.
30. Blank to be used is 0.1% FA (2 μl).
31. Spot 2 μl of samples onto the μDrop plate along with the blank.
32. Click plate layout → Select μDrop plate from the dropdown menu → Add details about the plate map
33. Click protocol → Absorbance → Multiple wavelengths → Add 205 and 280 nm.
34. Click results → blank subtraction
35. Run plate out → Place the μDrop plate in designated position → Run plate in → Start
36. Calculate E^205^ using the following formula: E^205^= 27/1-3.85^*^A^280^/A^205^.
  **CRITICAL:** The value of E205 should be 31 ± 3 mLmg^−1^cm^−1^. If value E205 is not lying in this range, the sample may not be properly digested.
37. Calculate conc. using formula: Absorbance (A^205^) = E^205*^ conc. ^*^ path length (0.05)
38. Conc. (μg/μl) = A^205^/ E^205^ (calculated from above formula) ^*^ path length (0.05)

## Experiment 1: Label-Free Quantitation (LFQ) of Plasma Sample Timing 2 h per Sample

39. The desalted peptides can be run for label-free quantitation using the below-mentioned LC (Section A) and MS (Section B) parameters. We observed good reproducibility between three technical replicates (see anticipated results below).

### A. LC Parameters

- Take 2 μg of digested peptides and make up the volume to 10 μl.
- **CRITICAL:** The concentration of desalted peptides will be 200 ng/μl.
- Place the vials in the auto-sampler stand of nLC 1200.
- Equilibrate the pre-column (Thermo Fisher Scientific, P/N 164564, S/N 10694527) and analytical column (Thermo Fisher Scientific, P/N ES803A, S/N 10918620) five times of column volume with 0.1% (v/v) FA.
- Load 1 μg of digested peptides onto the column using the nLC 1200 system.
- Set up the LC gradient based on sample complexity. We have used 120 min LC gradient for label-free quantitation of the plasma samples. The brief details of LC gradient are mentioned in Supplementary Method 1, 2.

### B. MS Parameters

- Open Thermo Scientific Xcalibur software double click on instrument setup and select the template from peptides-ID with default parameters (Figure S2).
- Populate the MS parameters from Figure S3 and save as a new method (Supplementary Method 3).
- Open Thermo Scientific Xcalibur software, double click on sequence setup and fill sample details such as sample type, sample name, file save location, instrument method file, the volume of injection, and position of the sample.
- Select the row and click on the run sequence.

## Experiment 2: Label-Based Quantification (iTRAQ 4-plex/ TMT 6-plex) of Plasma Sample Timing 5 h

40. The digested peptides can be labeled with iTRAQ, TMT reagents, etc. for label-based quantification. We have used iTRAQ 4-plex and TMT 6-plex for the labeling of digested peptides. The procedure for labeling and parameters of LC and MS is mentioned below.

### Labeling of Digested Peptides Using iTRAQ Reagents

- Allow each vial of iTRAQ® reagent required to reach room temperature (~5 min).
- Spin each vial (30 s) to bring the solution to the bottom of the vial.
- **CRITICAL:** Please check the vial. There should be a 10–15 μl solution.
- Add 70 μl of ethanol to each iTRAQ® Reagent vial.
- Vortex each vial to mix (30 s), and then spin (10 s).
- Transfer the contents of the iTRAQ® reagent vial to each sample tube (114, 115, 116, and 117). In this experiment, 12.5, 25, 50, and 100 μg of digested peptides labeled with the content of each vial's iTRAQ reagents 114, 115, 116, and 117, respectively.
- **CRITICAL STEP:** Organic part of the mixture should be >70%; if not, add more ethanol to keep it above 70%.
- Vortex each tube to mix (30 s), and then spin (10 s).
- **CRITICAL**: Check the pH by placing 1 μl of the solution on pH paper with a pH range of 8.0 to 10.0. If necessary, add up to 10 μl of dissolution buffer—plasma to adjust the pH to >8.
- Incubate at room temperature for 90 min.
- Add 100 μl of Milli-Q water to quench the reaction.
- **CRITICAL**: Ensure the aqueous part of the mixture >30%.
- Incubate the tubes at room temperature for 30 min.
- Combine the contents of all iTRAQ Reagent-labeled sample tubes into one tube.
- Vortex each tube to mix (30 s), and then spin (10 s).
- Dry the tube containing all the combined iTRAQ labeled peptides.

### LC Parameters

- Follow the steps 39Ai-39Avi from #experiment 1.
- Set up the LC gradient based on sample complexity. We have used a 180 min LC gradient for label-based quantitation (iTRAQ) of a plasma sample. The brief details are mentioned in the reagent set up.

### Generate an Instrument Method for iTRAQ Technique

- The MS parameters for label-based quantitation were the same, which were used for label-free quantitation except for collision energy. In the case of label-based quantitation, 35% collision energy was used for MS/MS fragmentation (Supplementary Method 3).

### Labeling of Digested Peptides Using TMT 6-Plex Reagents and Fractionation Using High-pH Reverse-Phase Technique

- Allow each vial of TMT 6-plex reagent to reach room temperature (~5 min).
- Spin each vial (2 min) to bring the solution to the bottom of the vial by occasional vortexing.
- Add 45 μl of anhydrous acetonitrile to each TMT 6-plex Reagent vial.
- Vortex each vial to mix (30 s), and then spin (10 s).
- Carefully add 40 μl of the TMT label reagent to each 50 μg of digested proteins.
- Vortex each tube to mix (30 s), and then spin (10 s).
- Incubate the reaction at room temperature for 90 min.
- Add 2 μl of 5% hydroxylamine to the sample and incubate for 30 min to quench the reaction.
- Combine the contents of all TMT reagent -labeled sample tubes into one tube.
- Vortex each tube to mix (30 s), and then spin (10 s).
- Dry the tube containing all the combined TMT labeled peptides.
- Fractionate the labeled peptide samples following manufacturer's instructions [Pierce™ High pH Reversed-Phase Peptide Fractionation Kit (Thermo Scientific™, cat no. 84868)].

### LC Parameters

- Follow the steps 39Ai-39Avi from experiment #1.
- Set up the LC gradient based on sample complexity. We have used a 90 min LC gradient for label-based quantitation (TMT 6-plex) of a plasma sample. The brief details of LC gradient are mentioned in Supplementary Method 1, 2.

### Generate an Instrument Method for TMT 6-plex Technique

- The MS parameters for label-based quantitation were the same, which were used for label-free quantitation except for collision energy. In the case of label-based quantitation, 35% collision energy was used for MS/MS fragmentation (Supplementary Method 3).

## Experiment 3: Multiple Reaction Monitoring (MRM) Assay Timing 1.5 h

41. The MRM assay was optimized using 21 heavy label synthetic peptides and then endogenous peptides were monitored using the optimized MRM protocol. The steps for method generation and parameters of LC and MS are mentioned below.

### Instrument Method Generation for MRM Using Skyline

- Load the sequence of synthetic peptides into Skyline and set the parameters for peptide and transition setting as mentioned in Figures S4, S5.
- Export the unscheduled transition list as a single method from Skyline (Figure S6A).
- Import the unscheduled transition list as an Inclusion list in a MRM acquisition method in Xcalibur.

### LC Parameters

- Follow the steps 39Ai-39Avi from #experiment.
- We have used 60 min LC gradient for Multiple Reaction Monitoring (MRM) of a plasma sample. The brief details of LC gradient are mentioned in Supplementary Methods 1, 2.

### Set Up Instrument Method for MRM

- Open Thermo Scientific Xcalibur software double click on instrument setup and select the template from the MRM template with default parameters.
- Import the unscheduled transition list as an Inclusion list in a MRM acquisition method in Xcalibur.
- Populate the MS parameters from Figure S7 and save as a new method.
- Open Thermo Scientific Xcalibur software double click on sequence setup and fill sample details such as sample type, sample name, file save location, instrument method file, the volume of injection, and position of the sample.
- Select the row and click on the run sequence.

## Experiment 4: Parallel Reaction Monitoring (PRM) Assay Timing 1.5 h

42. The PRM assay was optimized using 21 heavy label synthetic peptides, and then endogenous peptides were monitored using the optimized protocol. The steps for method generation and parameters of LC and MS are mentioned below.

### Instrument Method Generation for PRM Using Skyline

- Load the sequence of synthetic peptides into Skyline and set the parameters as mentioned in Figures S4, S5.
- Export the unscheduled isolation list as a single method from Skyline (Figure S6B).

### LC Parameters

- Follow the steps 39Ai-39Avi from experiment #1.
- We have used the same LC gradient for PRM, which we have used for MRM.

### Set Up Instrument Method for PRM

- Open Thermo Scientific Xcalibur software double click on instrument setup and select the template from MS^n^ with default parameters.
- Import the unscheduled isolation list as an Inclusion list in a Targeted-MS^2^ acquisition method in Xcalibur and populate the MS parameters from Figure S8 and save as a new method.
- Open Thermo Scientific Xcalibur software, double click on sequence setup and fill sample details such as sample type, sample name, file save location, instrument method file, the volume of injection, and position of the sample.
- Select the row and click on the run sequence.

## Data Analysis Timing Around 1 d

43. The proteomic data analysis of global and targeted experiments performed using different tools.

### Global Proteomics Data Analysis

- Raw instrument files were processed using Proteome Discoverer (PD) version 2.2 (Thermo Fisher Scientific). MS2 spectra were searched using the Sequest HT and Mascot (v2.6.0) search engine against *Homo sapiens* fasta (74,212 sequence entries, dated: 22/08/2019,) from Uniprot database (Proteome ID: UP000005640, Organism ID: 9606). All searches were configured with dynamic modifications for the iTRAQ reagents (+144.102 Da) on lysine and N-termini, and for TMT reagents (+229.163 Da) on lysine and N-termini of the peptide and oxidation of methionine residues (+15.9949 Da) and static modification as carbamidomethyl (+57.021 Da) on cysteine, monoisotopic masses, and trypsin cleavage (max 2 missed cleavages). The peptide precursor mass tolerance was 10 ppm, and MS/MS tolerance was 0.05 Da. The false discovery rate (FDR) for proteins, peptides, and peptide spectral matches (PSMs) peptides were kept 1%. The quantification values for proteins were exported from proteome discoverer 2.2. The brief parameters were mentioned in Table 1. The .raw files from the label-free method were searched against the same database. Most of the proteome discoverer parameters were kept the same as above mentioned for iTRAQ 4-plex method except dynamic modifications for the iTRAQ reagents (+144.102 Da) on lysines and N-termini of a peptide and for TMT reagents (+229.163 Da) on lysine and N-termini of the peptide.
- We normalized the data sets using the abundance of total peptide for the identification of differentially expressed proteins. The normalization by total peptide amount is the default option in Proteome Discoverer (v2.2). In this case, it sums the peptide group abundances for each sample and determines the maximum sum for all files, and it calculates the normalization factor using the sum of the sample and the maximum sum in all files.

**Table 1:** The brief details of proteome discoverer parameters.

**Description**	**Parameters**	**Description**	**Parameters**
**Processing Workflow**		**Consensus Workflow**	
Spectrum Files RC		Feature mapper	
Precursor Mass Tolerance	20 ppm	Maximum RT shift	5
Fragment mass tolerance	0.05 Da	Mass tolerance	10
Enzyme name	Trypsin	Peptide validator	
**Mascot**		Validation mode	Only PSM level FDR calculation base
Enzyme name	Trypsin	Target FDR (Strict) for PSMs	0.05
Maximum missed cleavage	2	Target FDR (Relaxed) for PSMs	0.01
Precursor Mass Tolerance	10 ppm	Target FDR (Strict) for Peptide	0.01
Fragment mass tolerance	0.05 Da	Target FDR (Relaxed) for Peptide	0.05
Dynamic modification	**LFQ:** Oxidation (M), Phospho (STY); **iTRAQ:** iTRAQ 4plex, Oxidation (M), Phospho (STY); **TMT:** TMT 6-plex, Oxidation (M), Phospho (STY)	Validation based on	q-value
Static modification	Carboamidomethyl (C)	Use concatenated	TRUE
**Sequest**		Precursor ions quantifier	
Enzyme name	Trypsin	Normalization mode	Total peptide
Maximum missed cleavage	2	Scaling mode	On all average
Precursor Mass Tolerance	10 ppm	Imputation mode	Low abundance resampling
Fragment mass tolerance	0.05 Da	Protein marker	
Dynamic modification	**LFQ:** Oxidation (M), Acetyl (N-Terminus), Phospho (STY); **iTRAQ:** iTRAQ 4plex (K and N-Terminal), Oxidation (M), Acetyl (N-Terminus), Phospho (STY); **TMT:** TMT 6-plex (K, N-Terminal), Oxidation (M), Phospho (STY)	Protein database	Contaminants-23042018
Static modification	Carboamidomethyl (C)	Results	
Minimum peptide length	6	Protein	Master
Percolator		Protein confidence	High
Target FDR (Strict)	0.01	Unique protein	Depends on data
Target FDR (Relaxed)	0.05		

The users may also use additional data normalization in subsequent steps. There are several normalization approaches, including central tendency, linear regression, locally weighted regression, quantile techniques, and others (33). The normalization methods are evaluated in terms of their ability to reduce variation between technical replicates. Although all these methods can reduce the systematic bias to some extent, each approach has its own advantages and disadvantages (33–35). Therefore, the selection of the normalization approaches also depends on the experimental designs and type of data sets.

### Targeted Proteomics Data Analysis

The steps for data analysis of MRM and PRM are the same. We have performed data analysis using Skyline (Skyline-daily 19.1.9.350).

- Open the skyline document containing the list of transitions.
- Now click on import results located under the file tab as shown in Figure S9A.
- Locate the folder containing the results and upload the files at once. You would see a window like the one shown in Figure S9B.
- Once the import is completed, look at the retention times of the peaks that Skyline detects automatically. To ensure that the right peak has been detected, go to the “View” tab and select replicate comparison under the retention time option.
- Now correct the retention times of peptides that have been wrongly annotated by Skyline.
- **CRITICAL STEP:** Consider the dot *p* values, shape and intensity of the peak among the many other parameters while deciding on the right peak. The re-annotation involves dragging the mouse cursor below the X-axis from the start time to the end time of the eluted peak.
- Once the re-annotation is complete and the areas of all the peaks have been corrected, save the document.
- Export the data and perform statistical data analysis.

## Timing

Steps 1-2, Plasma sample preparation: 20 min
Steps 3-9, Depletion of high abundant proteins: 1 h
Steps 10-14, Protein quantification & sample preparation for digestion: 2 h
Steps 15-17, Enzymatic digestion of plasma proteins: 6-8 h
Steps 18-27, Desalting of digested peptides: 1 h per 5 samples
Steps 28-38, Quantification of desalted peptides: 10 min
Steps 39A-39B, Experiment 1: Label-free quantitation (LFQ) of plasma sample: 2 h
Steps 40A-40F, Experiment 2: Label-based quantitation (iTRAQ 4-plex/ TMT 6-plex) of plasma sample: 5 h
Steps 41A-41C, Experiment 3: Multiple Reaction Monitoring (MRM) assay: 1.5 h
Steps 42A-42C, Experiment 4: Parallel Reaction Monitoring (PRM) assay: 1.5 h
Steps 43A-43B, Data analysis: around 1 d

**Troubleshooting**

**Table:** 

**Steps**	**Problem**	**Possible Reason**	**Solution**
Step 9	Incomplete removal of albumin or IgG	Sample exceeds binding capacity	Reduce the amount of sample processed
		Incomplete binding	Increase incubation time
		The sample is not mixed properly during incubation	Mix the sample with resin by gentle end-over-end mixing and make sure that the sample is mixing with the resin during the incubation period
Step 15–17	Incomplete digestion of proteins	pH is not adjusted to ~8	Adjust the pH of the sample by adding 100 mM ammonium bicarbonate.
		Urea concentration can be more than >1 M	Dilute the sample by adding 100 mM ammonium bicarbonate.
Step 18	Too much background	Buffer, salt interference	Clean-up sample with desalting column (Empore C18 disk, Pierce C18, Spin columns)
		Keratin contamination	Use fresh buffer and always wear gloves
Step 40	Less no. of proteins identified in case of iTRAQ/TMT	Less amount of peptide considered for labeling	Label at least 50 μg of each iTRAQ/TMT reagent
		Labeling efficiency is not good	Check the pH before adding iTRAQ/TMT reagents and incubate it 2 h with intermittent vortexing after every 10 min
Steps 40	Chromatogram is not good	LC setting	Optimize LC setting for your sample
		Salt and other contaminants	Clean-up sample with desalting column (Empore C18 disk, Pierce C18, Spin columns)
		Column issue	Replace the column
Step 40	Fragmentation of the peptides is not good	Poor ionization	Clean the front end of the mass spectrometer
		Bubble issue at the tip of column	Increase the voltage till 2.3 kV, if not resolved, change the analytical column

## Results

One of the major challenges of cancer plasma proteomics has been its inability to discover markers with clinical implications. However, improvement in instrumentation and mass spectrometry-based platforms have contributed to the revival of plasma proteomics (36–39). Currently, several proteomics techniques are being used for MS-based quantitation of plasma proteins for different cancer projects. This study provides a complete proteomics workflow for the discovery and validation of potential biomarker candidates from plasma samples using mass spectrometry. Additionally, the study also provides an optimized sample preparation strategy to get decent coverage of the plasma proteome, which is essential for cancer biomarker discovery projects.

**Figure 2:**
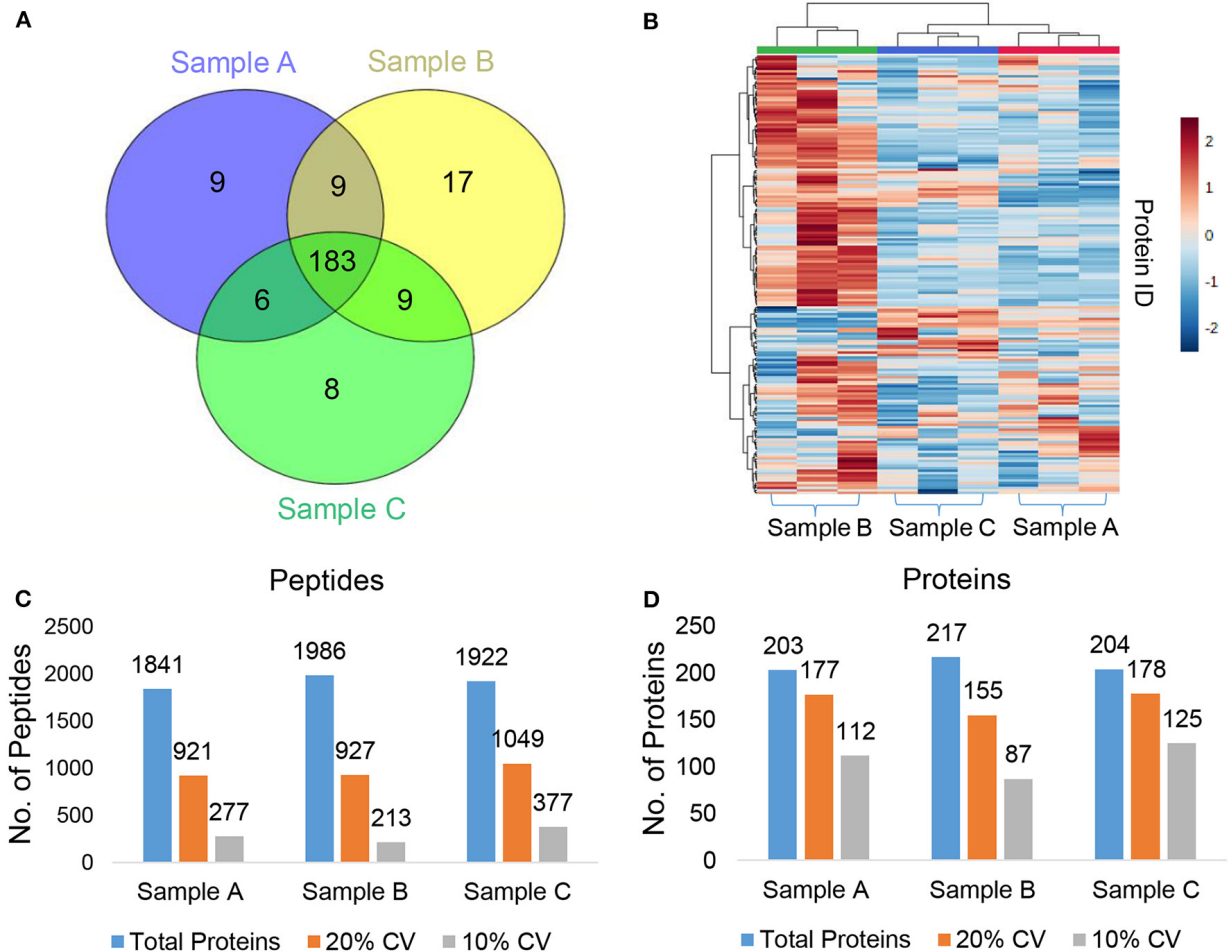
Label-free quantitation (LFQ) of plasma proteins. **(A)** Venn diagram representing the common and unique proteins across the different plasma samples. **(B)** Heatmap showing abundances of the identified proteins in each technical replicate of the three pooled plasma samples (Sample A, B, and C). **(C,D)** The total number of identified **(C)** peptides **(D)** proteins, no. of proteins below 20%, and 10% coefficient of variation (CV) at 1% FDR.

**Figure 3:**
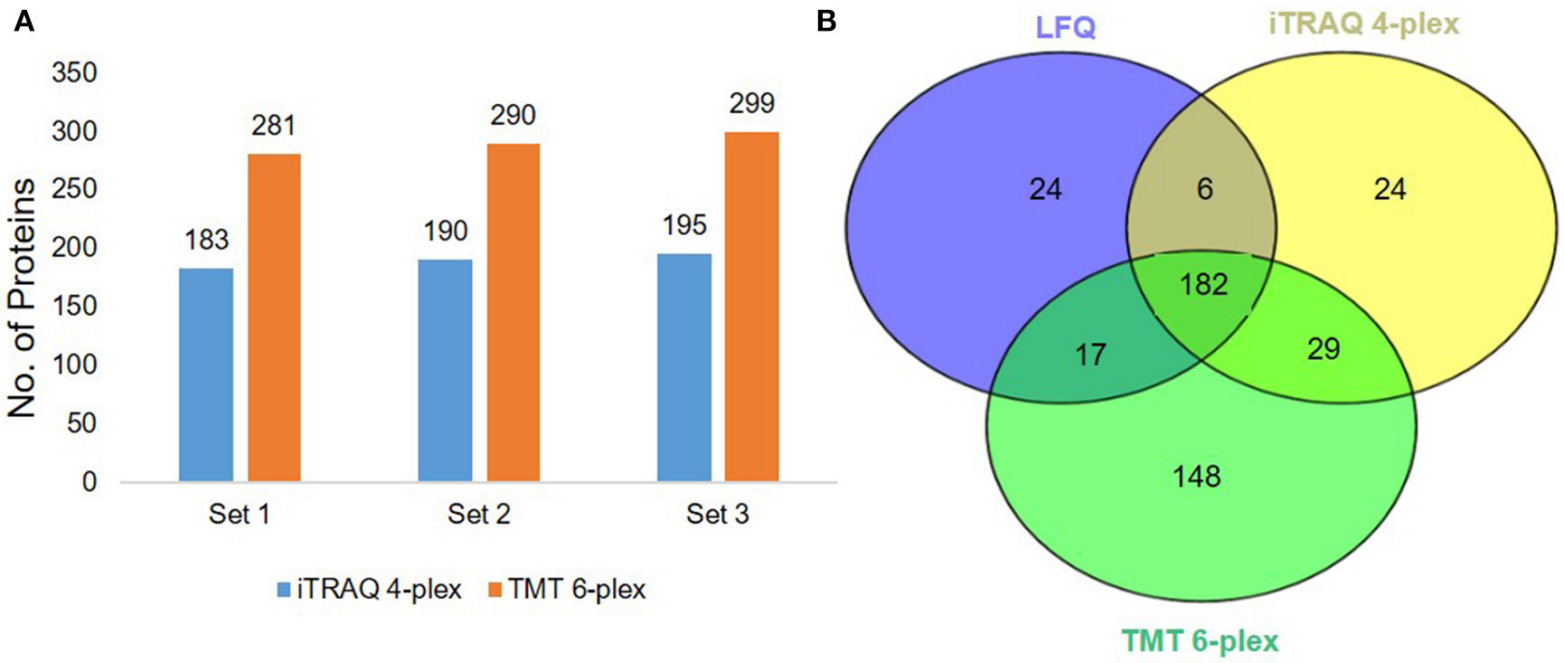
Label-based quantitation of plasma proteins. **(A)** The total no. of identified proteins iTRAQ 4-plex and TMT 6-plex experiments at 1% FDR. **(B)** Venn diagram representing the common and unique proteins across the three different approaches i.e., label-free quantitation, iTRAQ 4-plex, and TMT 6-plex.

We used a 120 min LC gradient for label-free quantitation and (Figure S10A) detected 2332 peptides corresponding to 241 proteins with at least one unique peptide at 1% FDR (Table S1). We identified 183 proteins common in all three samples (Figure 2A). The heatmap and correlation matrix indicate high levels of consistency (Pearson *r* value > 0.99) (Figure 2B and Figure S10B) between the technical replicates (R1, R2, and R3) of different biological samples (Sample A, B, and C). We observed an average of 965 peptides and 170 proteins below than 20% coefficient of variation (CV) (Figures 2C,D). In case of iTRAQ experiment, we have labeled varying amounts of peptides (100, 50, 25, 12.5 μg) using iTRAQ reagents to check the minimum amount of peptide to be labeled and observed minimum 50 μg amount of peptide could be used for the good quantitative proteomics experiment (Figure S11). However, the number of proteins identified in 114 labeled samples was relatively lower than the other three labels, i.e., 115, 116, and 117. This could be a result of labeling a significantly low number of peptides with the 114-label compared to the other three labels. Around 219 proteins were identified and quantified using iTRAQ-based multiplexed quantitative proteomics (Figure 3A, Table S2). In TMT experiments, we identified 376 proteins, and 182 proteins were common across all the three quantitative proteomics techniques (LFQ, iTRAQ 4-plex, and TMT 6-plex) (Figure 3B, Table S3). Studies performing in-depth comparisons of label-free and label-based quantitation (37, 40–42) are also available. We observed a slight increase in the identification of proteins using fractionated samples (six fractions) of TMT 6-plex experiment in comparison to label-free quantitation and iTRAQ 4-plex with a 43.3% overlap between the proteins identified using all three approaches (Figure 3B). Further, LFQ provides the flexibility of analyzing clinical samples processing and running as or when available and generating individual datasets. Obtained peptides/protein datasets could be analyzed in different contexts based on IHC, radiology, and other known clinical parameters to address various clinical questions in cancer biology.

**Figure 4:**
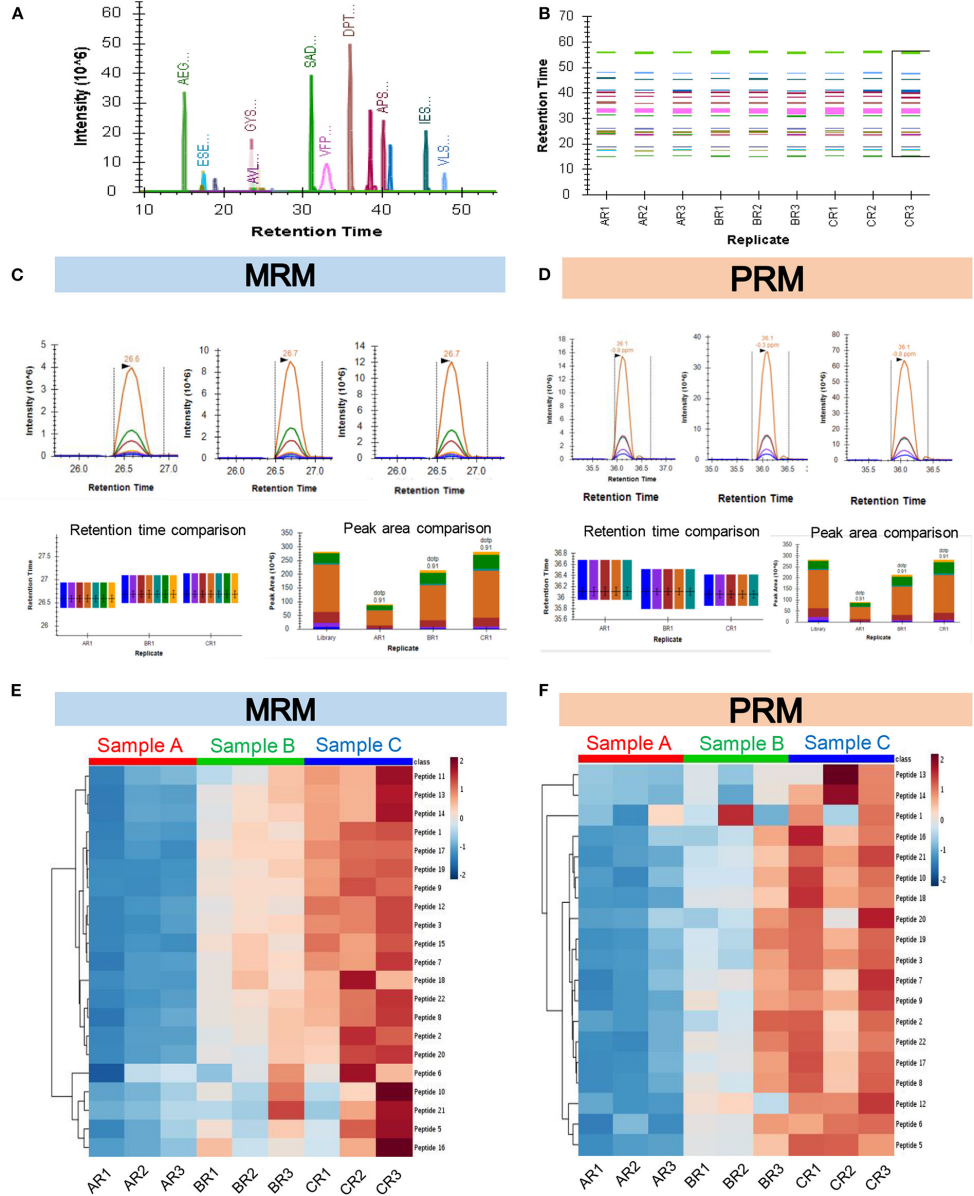
Quantification of synthetic peptides using targeted MS approaches. **(A)** Representative image for all synthetic peptides quantified in the PRM experiment. The plot shows the retention times of the peptides along the x-axis vs. their corresponding intensities along the y-axis. **(B)** Plot showing consistency in retention times of the peptides across all the replicates. **(C)** Representative peak intensities, Retention times, and Peak areas across various dilutions for the Peptide DPTFIPAPIQAK as observed in the MRM experiment. **(D)** Representative peak intensities, Retention times, and Peak areas across various dilutions for the Peptide DPTFIPAPIQAK as observed in the PRM experiment. **(E,F)** The heatmap showing abundances of the identified proteins in each technical replicate (R1, R2, R3) for three biological pools of plasma samples in increasing order of concentration (A, B, and C) as seen in the MRM **(E)** and PRM **(F)** experiments.

**Figure 5:**
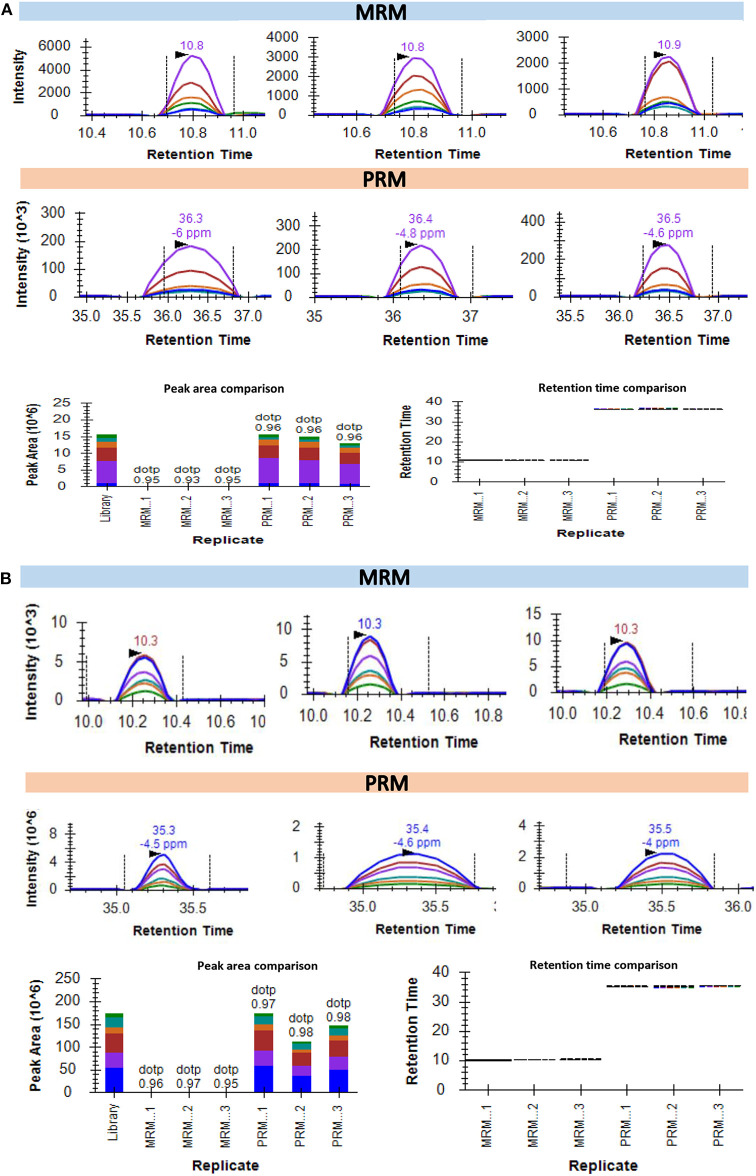
Quantification of the endogenous peptides in plasma samples using targeted MS approaches. **(A)** Intensities of AGALNSNDAFVLK for protein Gelsolin-1 in 3 plasma samples as detected using MRM and PRM. The bottom left part of the panel represents the comparative peak areas of the peptide in each of the 3 samples using both the techniques. The bottom right of the panel shows the consistency of retention times across the biological replicates in both the techniques. **(B)** Intensities of SGLSTGWTQLSK for protein Alpha-1B-glycoprotein in 3 plasma samples as detected using MRM and PRM. The bottom left part of the panel representing the comparative peak areas of the peptide in each of the 3 samples using both the techniques. The bottom right of the panel shows a comparative analysis.

The recent developments in the field of targeted proteomics are showing promises in bridging the gap between biomarker discovery and validation of the potential biomarkers (15, 30, 43). We have provided here a workflow for targeted proteomics using PRM and MRM approaches. The main difference between PRM and MRM is that we have to define the transition list in case of MRM and isolation list in case of PRM (Figure S12). The abundance of each peptide and reproducibility of retention time across three technical replicates of three biological samples are represented in Figures 4A,B. The representative peak intensities, retention times and peak areas across various dilutions for the peptide DPTFIPAPIQAK as observed in the MRM experiment and PRM experiment (Figures 4C,D). The intensity of the synthetic peptides using MRM and PRM was found to be correlated with the levels of synthetic peptides spiked into samples A, B, and C (Figures 4E,F). We monitored the levels of a few potential cancer biomarkers in plasma samples using MRM, and PRM approaches. The peptide AGALNSNDAFVLK from Gelsolin-1 and SGLSTGWTQLSK from Alpha-1B-glycoprotein showed a good response (Intensities of 10^3^ in MRM and 10^6^ PRM) and good spectral match with library (dotp value > 0.93) in both the targeted approaches (Figures 5A,B).

## Discussion

Quantitative approaches involving ultra-sensitive mass spectrometers, which are presented as the pinnacle of promising proteomics technologies, are undoubtedly one of the most widely used approaches in biomarker discovery in recent years. The integrated quantitative proteomics pipeline combining global and targeted approaches described here could be extremely useful in cancer biomarker discovery and validation in plasma samples without a need for any separate immunoassay-based validation method.

Preanalytical variables introduced during blood collection, plasma separation, and storage conditions can adversely influence the quantification of proteins in plasma samples (44), and thereby the outcome of the overall analysis. Potential cancer biomarkers are often very low-abundance proteins and the numbers of detectable proteins are restricted by the complexity of plasma or serum proteome (6, 45, 46). Therefore, it requires extensive depletion of the high-abundance proteins and fractionation methods to obtain comprehensive coverage of the plasma proteome, which certainly introduces substantial experimental time and cost in such quantitative proteomics workflow. In general, the establishment of any clinically relevant protein biomarker panel requires analysis involving large clinical cohorts, including multiple types of control populations (2, 23), which is more crucial for cancer biomarker based projects due to the inter- and intra-tumoral heterogeneity. However, the sample throughput of the discovery phase quantitative proteomics is still moderate and not adequately efficient to satisfy this need (47). Finally, due to the requirement of sophisticated instrumentation and experienced personnel, such MS-based quantitative proteomics workflow is not suitable for routine screening of blood samples in clinical setups.

Analysis of plasma proteome using two complementary quantitation methods as described here provided a satisfactory coverage. Despite advancements in biomarker discovery, there is still no consensus on whether pooling serum samples for shotgun proteomics experiments is always advisable in the discovery phase. While there are many studies that have used serum pooling as a strategy for cancer biomarker discovery (48–51), there also exist studies which advocate otherwise (52, 53). Pooling of clinical samples are often practiced in quantitative proteomics analysis when large numbers of samples need to be studied or there is not an adequate amount of each sample for individual analysis. If sample pooling is performed during the discovery phase of the analysis, it is essential to validate the results in individual diseased and control samples selected randomly from the pooled populations.

In this workflow, the discovery phase experiments [Label-free (LFQ) and Label-based (iTRAQ or TMT)] were performed using an Orbitrap Fusion instrument. The targeted (validation) experiments were performed using two different platforms: multiple reaction monitoring (MRM) using a Triple Quadrupole instrument, and parallel reaction monitoring (PRM) using an Orbitrap Fusion instrument. These two techniques are based on similar principles, and the choice of the method is largely reliant on the type of instrument available to the users. Plasma abundance of a potential cancer biomarker—Alpha-1B-glycoprotein was monitored in the pooled samples and further validated in individual samples using MS-based targeted approaches (Figure S13). Using this integrated quantitative proteomics workflow we were able to quantify the relatively low abundant plasma proteins as well (Figure S14). The targeted approaches were found to be much superior in terms of quantification accuracy in comparison to the shotgun proteomics approaches. While MRM experiments can be carried out on low-resolution instruments like the triple quadrupole LC-MS (QqQ LC-MS), PRM experiments require the use of high-resolution LC-MS instruments with the QTOF or Q-Orbitrap configuration. Taken together, we conclude that plasma proteomics-based cancer biomarker projects could heavily benefit from detailed workflows of quantitative and targeted proteomics provided in this study. We have demonstrated here multiple possible quantitative approaches in the discovery and validation phases of this combined workflow, but all the methods are not required to be performed simultaneously. Different combinations including any of these discovery and validation phase approaches, could be implemented in biomarker research. Selection of the specific label-based or label-free quantification approach for discovery workflow and MRM or PRM for targeted workflow may depend on the key biological question to be addressed, number of samples, and availability of MS instruments and resources.

## Data Availability Statement

The datasets presented in this study can be found in online repositories. The names of the repository/repositories and accession number(s) can be found below: https://www.ebi.ac.uk/pride/archive/, PXD017834, http://www.peptideatlas.org/ (54), PASS01619.

## Ethics Statement

The studies involving human participants were reviewed and approved by institutional review boards and ethics committee of the Indian Institute of Technology Bombay (IITB-IEC/2016/026). The patients/participants provided their written informed consent to participate in this study.

## Author Contributions

VK, SS, and SR conceived and designed the experiments. VK performed the MS-based quantitative proteomics experiments and data were analyzed by VK, SR, and SG. The manuscript was written by VK, SR, SG, and SS. All authors agreed on the interpretation of data and approved the final version of the manuscript.

## Conflict of Interest

The authors declare that the research was conducted in the absence of any commercial or financial relationships that could be construed as a potential conflict of interest.
