# Supplementary material for: An Integrated Quantitative Proteomics Workflow for Cancer Biomarker Discovery and Validation in Plasma
Source: Front Oncol. 2020 Sep 23;10:543997. doi: 10.3389/fonc.2020.543997 (PMC7538778; doi:10.3389/fonc.2020.543997)
Supplement: Supplementary file 1 [file Data_Sheet_1.PDF]

Supplementary Materials for

An Integrated Quantitative Proteomics Workflow for Cancer Biomarker Discovery and Validation in Plasma

Vipin Kumar[1], Sandipan Ray[1, 2], Saicharan Ghantasala[1], Sanjeeva Srivastava[1]*

[1]Department of Biosciences and Bioengineering, Indian Institute of Technology Bombay, Mumbai 400076, India

[2]Present address: Department of Systems Pharmacology and Translational Therapeutics, Perelman School of Medicine, University of Pennsylvania, Philadelphia, PA 19104, USA

[*]Correspondence: Dr. Sanjeeva Srivastava, E-mail: sanjeeva@iitb.ac.in

Phone: +91-22-2576-7779, Fax: +91-22-2572-3480

This file includes

Supplementary method 1

Supplementary method 2

Supplementary method 3

Supplementary Figures

Figure S1 | Steps involved in the preparation of the C18 desalting tip.

Figure S2 | Creating a data-dependent method using Thermo Xcalibur software.

Figure S3 | LC and MS parameters used for label-free quantitation.

Figure S4 | Peptide settings for Multiple Reaction Monitoring and Parallel

Reaction Monitoring using Skyline.

Figure S5 | Transition settings for Multiple Reaction Monitoring and Parallel

Reaction Monitoring using Skyline

Figure S6 | (A, B) Procedure for exporting the list of peptides for Multiple Reaction Monitoring **(A)** and Parallel Reaction Monitoring **(B)** experiment.

Figure S7 | Method Parameters for Multiple Reaction Monitoring experiment on TSQ Altis.

Figure S8: Method parameters for the Parallel Reaction Monitoring experiment on Orbitrap Fusion.

Figure S9 | Data analysis using Skyline. **(A)** Importing the result files into the Skyline document. **(B)** Steps to view the retention time of peptides.

Figure S10 | (A) The gradient used for a 2-hour separation through liquid chromatography. **(B)** Correlation analysis of the technical replicates of three different biological pools of plasma samples.

Figure S11 | The total no. proteins which has a coefficient of variation of less than 20% and 10% in each of the iTRAQ 4-plex labels- 114, 115, 116, and 117 after applying 1% FDR. The number of labelled peptides varied across the iTRAQ reagents.

Figure S12 | Targeted proteomics using MS-based approaches. **(A, B)** Workflow for Multiple Reaction Monitoring (MRM), this approach considers the transitions of the peptide **(A)** and Parallel Reaction Monitoring (PRM), this approach considers only selection of peptides **(B)**. It is independent of transitions.

Figure S13 | The abundance profile of alpha-1B-glycoprotein in the pooled samples (iTRAQ experiment) and individual samples (TMT, MRM, and PRM experiments) using different proteomic techniques. The y-axis represents the abundance in Log_2, and the x-axis represents samples used in iTRAQ, TMT, MRM, and PRM experiments. The protein abundance of alpha-1B-glycoprotein is the average abundance of three peptides (R.SGLSTGWTQLSK.L, R.CEGPIPDVTFELLR.E, and R.TPGAAANLELIFVGPQHAGNYR.C) in case of MRM and PRM experiments. P: Pooled sample, S: individual sample.

Figure S14 | The abundance profile of vascular cell adhesion protein 1 in label-free quantitation (LFQ), iTRAQ-4plex, TMT-6plex, and MRM experiments. The y-axis represents the abundance in Log_2, and the x-axis represents samples used in LFQ, iTRAQ-4plex, and TMT-6plex experiments. S: Plasma sample.

Supplementary Tables

Table S1 | (Microsoft Excel format). The list of identified proteins in label-free quantitation experiment.

Table S2 | (Microsoft Excel format). The list of identified proteins in iTRAQ 4-plex experiment.

Table S3 | (Microsoft Excel format). The list of identified proteins in TMT 6-plex experiment.

Supplementary method 1

Material

Biological Materials

1. Blood

CAUTION In this study, the collection of blood samples was done at the Calcutta Medical College, Kolkata, India. Prior to the sample collection process, written informed consent was received from each participant after giving detailed explanations about the experimental procedure in the language best understood by the potential participants. This study was approved by the institutional review boards and ethics committee of the Indian Institute of Technology Bombay (IITB-IEC/2016/026). Human Body fluids should be handled in a Biosafety level 2 environment.

Reagents

1. Pierce™ Top 12 Abundant Protein Depletion Spin Columns (Thermo Fisher Scientific, cat no. 85165)
2. Quick Start Bradford Protein Assay Kit (BioRad, cat. no. 5000205) **CRITICAL** Please go through the manufacturer's instruction to check the compatibility of the reagent.
3. Urea (Sigma-Aldrich, cat. no. U0631) **CAUTION** It is a health hazard level 2 compound. Use gloves and avoid direct contact with skin and eyes.
4. Ammonium bicarbonate (Sigma-Aldrich, cat. no. 09830)
5. Tris (2-carboxyethyl) phosphine hydrochloride solution, 0.5 M, pH 7.0 (aqueous solution; pH was adjusted with ammonium hydroxide) (Sigma life Science, cat. no. 646547-10X1ML) **CAUTION** It is health hazard level 2, 2A and 3 compounds**.** Use gloves and avoid direct contact with skin and eyes.

6. Iodoacetamide (Sigma-Aldrich, cat. no. A3221)
7. Pierce™ trypsin protease, MS Grade (Thermo Fisher Scientific, cat. no. 90057)
8. Empore™ Octadecyl C18 47mm Extraction Disks (cat no. 66883-U)
9. iTRAQ® Reagents – 4-plex (SCIEX, PN 4374321)
10. TMT 6-plex™ Isobaric Label Reagent Set, 1 x 0.8 mg (Thermo Scientific™, cat no. 90061)
11. Pierce™ High pH Reversed-Phase Peptide Fractionation Kit (Thermo Scientific™, cat no. 84868)
12. Acetonitrile: Optima™ LC/MS grade (cat. no. A9554) **CAUTION** Flammable liquid and vapor. Irritating to eyes. May cause skin and respiratory tract irritation.
13. Methanol: Optima™ LC/MS grade, (cat. no. A456-500) **CAUTION** Flammable liquid and vapor. Irritating to eyes. May cause skin and respiratory tract irritation.
14. Formic Acid: LC-MS Ultra (Sigma-Aldrich; cat. no. 14265)

Equipment

1. Mini_PROTEAN® Tetra Cell and Systems (BioRad, cat no. 1658000)
2. Liquid chromatography: EASY-nLC 1200 (Thermo Fisher Scientific)
3. Mass spectrometer: Orbitrap Fusion and TSQ Altis (Thermo Fisher Scientific)
4. Pre-column: Acclaim™ PepMap™ 100 C18 HPLC Columns, 5 µm, 100 µm I.D. x 2 cm, nanoViper (Thermo Fisher Scientific, P/N 164564, S/N 10694527)
5. Analytical Column: PepMap™ RSLC C18, 2 µm, 75 µm x 50 cm (Thermo Fisher Scientific, P/N ES803A, S/N 10918620)
6. Thermo Scientific SPEEDVAC Savant SpeedVac Kit ISS110 P1
7. SmartSpec™ Plus Spectrophotometer (BioRad, cat. no. 170-2525)
8. Multiskan GO (Thermo Fisher Scientific, cat. no. N10588)

9. Micro-centrifuge capable of operating at 1000 ×g
10. End-over-end mixer
11. BD Vacutainer® PST™ (cat no. 367960)

Reagent setup

1. Plasma digestion buffer: Prepare a 100 mm ammonium bicarbonate solution at pH 8.0 by adding 3.953 g of ammonium bicarbonate to 1 l of milli-Q-water.
2. 6 M urea: Prepare 6 M urea by adding 3.6 g of urea to 10 ml of 100 mm ammonium bicarbonate buffer.
3. Solvent for desalting column, Solvent 1, for eluting the peptides, is 40% (v/v) acetonitrile.
   Solvent 2, for eluting the peptides, is 50% (v/v) acetonitrile.
   Solvent 3, for eluting the peptides, is 60% (v/v) acetonitrile.
4. Solvents for nLC, Solvent A 0.1% (v/v) formic acid in water, Solvent B 80% (v/v) acetonitrile in 0.1% (v/v) formic acid

Supplementary method 2

Chromatography Gradients

The liquid chromatography gradient used for label-free quantitation (LFQ) experiment is shown below. It summarizes the time duration for solvent B at different intervals of time with flow rate of 300 nl/min (Step 39).

Time [mm:ss]	Duration [mm:ss]	Flow [nl/min]	Mixture [%B]
**00:00**	00:00	300.00	02.00
**05:00**	05:00	300.00	05.00
**75:00**	70:00	300.00	25.00
**105:00**	30:00	300.00	45.00
**115:00**	10:00	300.00	95.00
**120:00**	05:00	300.00	95.00

The liquid chromatography gradient used for the label-based quantitation (iTRAQ 4-plex) experiment is shown below. It summarizes the time duration for solvent B at different intervals of time with flow rate of 300 nl/min (Step 40).

Time [mm:ss]	Duration [mm:ss]	Flow [nl/min]	Mixture [%B]
**00:00**	00:00	300.00	00.00
**10:00**	10:00	300.00	05.00
**120:00**	110:00	300.00	30.00
**170:00**	50:00	300.00	65.00
**175:00**	05:00	300.00	90.00
**180:00**	05:00	300.00	90.00

The liquid chromatography gradient used for the label-based quantitation (TMT 6-plex) experiment is shown below. It summarizes the time duration for solvent B at different intervals of time with flow rate of 300 nl/min (Step 40).

Time [mm:ss]	Duration [mm:ss]	Flow [nl/min]	Mixture [%B]
**00:00**	00:00	300.00	00.00
**04:00**	04:00	300.00	05.00
**62:00**	58:00	300.00	25.00
**80:00**	18:00	300.00	50.00
**83:00**	03:00	300.00	90.00
**90:00**	07:00	300.00	90.00

The liquid chromatography gradient used for Multiple Reaction Monitoring (MRM) and Parallel Reaction Monitoring (PRM) experiment is shown below. It summarizes the time duration for solvent B at different intervals of time with flow rate of 300 nl/min (Step 41 and 42).

Time [mm:ss]	Duration [mm:ss]	Flow [nl/min]	Mixture [%B]
**00:00**	00:00	300.00	02.00
**05:00**	05:00	300.00	10.00
**42:00**	37:00	300.00	35.00
**52:00**	10:00	300.00	45.00
**55:00**	03:00	300.00	90.00
**60:00**	05:00	300.00	90.00

Supplementary method 3

OT-HCD-OT MS/MS Method The following MS parameters were used for the LFQ experiment (Step 39). The same parameters were used for the iTRAQ 4-plex/TMT 6-plex experiment instead of collision energy. In case of the iTRAQ 4-plex/TMT 6-plex MS method, the collision energy was set 35% (Step 40).

Method settings
Application mode: Peptide
Method duration (min): LFQ:120 mins, iTRAQ 4-plex:180 mins, TMT 6-plex: 90 mins/fraction

NSI source/gas parameters
Spray voltage: 1900 V
Capillary temperature: 275°C
Sheath gas: 0
Auxiliary gas: 0

MS global settings
Default charge state: 1
Internal mass calibration: Lock Mass (445.12003 m/z)

Experiment #1 [MS]
Start time (min): 0
End time (min): 120
Cycle time (sec): 3

MS OT
Detector type: Orbitrap
Resolution: 60,000
Mass range: Normal

Use quadrupole isolation: True

Scan range (m/z): 375-1700

RF lens (%): 60

AGC target: 4.0e5

Maximum injection time (ms): 50

Microscans: 1

Data type: Profile

Polarity: Positive

NCE: 28

Lock masses: 445.12003 m/z

Monoisotopic peak determination: Peptide

Charge state

Include charge state (s): 2-6

Include undetermined charge states: False

Include charge states 25 and higher: False

Dynamic exclusion

Exclude after n times: 1

Exclusion duration (s): 40

Mass Tolerance: ppm

Low: 10

High: 10

Exclude Isotopes: True

Perform a dependent scan on single charge state per precursor only: False

Intensity

Filter Type: Intensity Threshold

Intensity Threshold: 5.0e3

Data Dependent

Data Dependent Mode: Cycle Time

Time between Master Scans (sec): 3

ddMS^2 OT HCD

Isolation Mode: Quadrupole

Isolation Window (m/z): 1.2

Isolation Offset: Off

Activation Type: HCD

Collision Energy (%): LFQ: 30, iTRAQ 4-plex/TMT 6-plex: 35

Detector Type: Orbitrap

Scan Range Mode: Auto: m/z Normal

Orbitrap Resolution: LFQ: 15000, iTRAQ 4-plex and TMT 6-plex: 30000

First Mass (m/z): 100

AGC Target: 1.0e4

Inject Ions for all available parallelizable time: True

Maximum Injection Time (ms): 30

Microscans: 1

Data Type: Centroid

Use EASY-IC^{tm}: False

The following MS parameters were used for Multiple Reaction Monitoring (Step 41).

NSI Source/Gas parameters

Spray Voltage: 2200 V

Capillary Temperature: 300 °C

Sheath Gas: 0

Auxiliary Gas: 0

MRM parameters

Use cycle time (sec): 3

Use calibrated RF lens: True

Q1 Resolution (FWHM): 0.7

Q3 Resolution (FWHM): 0.7

CID Gas (mTorr): 2

Source Fragmentation (V): 0

Chromatographic Peak Width (sec): 30

Use Chromatographic Filter: False

Use retention time reference; False

Display retention time: false

Use quan ion: False

Show visualization: false

The following MS parameters were used for Parallel reaction monitoring (Step 42).

NSI Source/Gas parameters

Spray Voltage: 1900 V

Capillary Temperature: 275 °C

Sheath Gas: 0

Auxiliary Gas: 0

tMS^2 OT HCD

MS^n Level (n): 2

Multiplex Ions: False

Isolation Mode: Quadrupole

Isolation window (m/z): 1.2

Activation Type: HCD

HCD collision energy (%): 30

Stepped collision energy: false

Detector type: Orbitrap

Orbitrap resolution: 60000

Mass range: normal

Scan range (m/z): 350-1500

RF lens (%): 60

AGC Target: 1.0e5

Inject Ions for all available parallelizable time: False

Maximum Injection Time (ms): 118

Microscans: 1

Data Type: Centroid

Polarity: Positive

Use EASY-IC^{tm}: False

Loop control: All

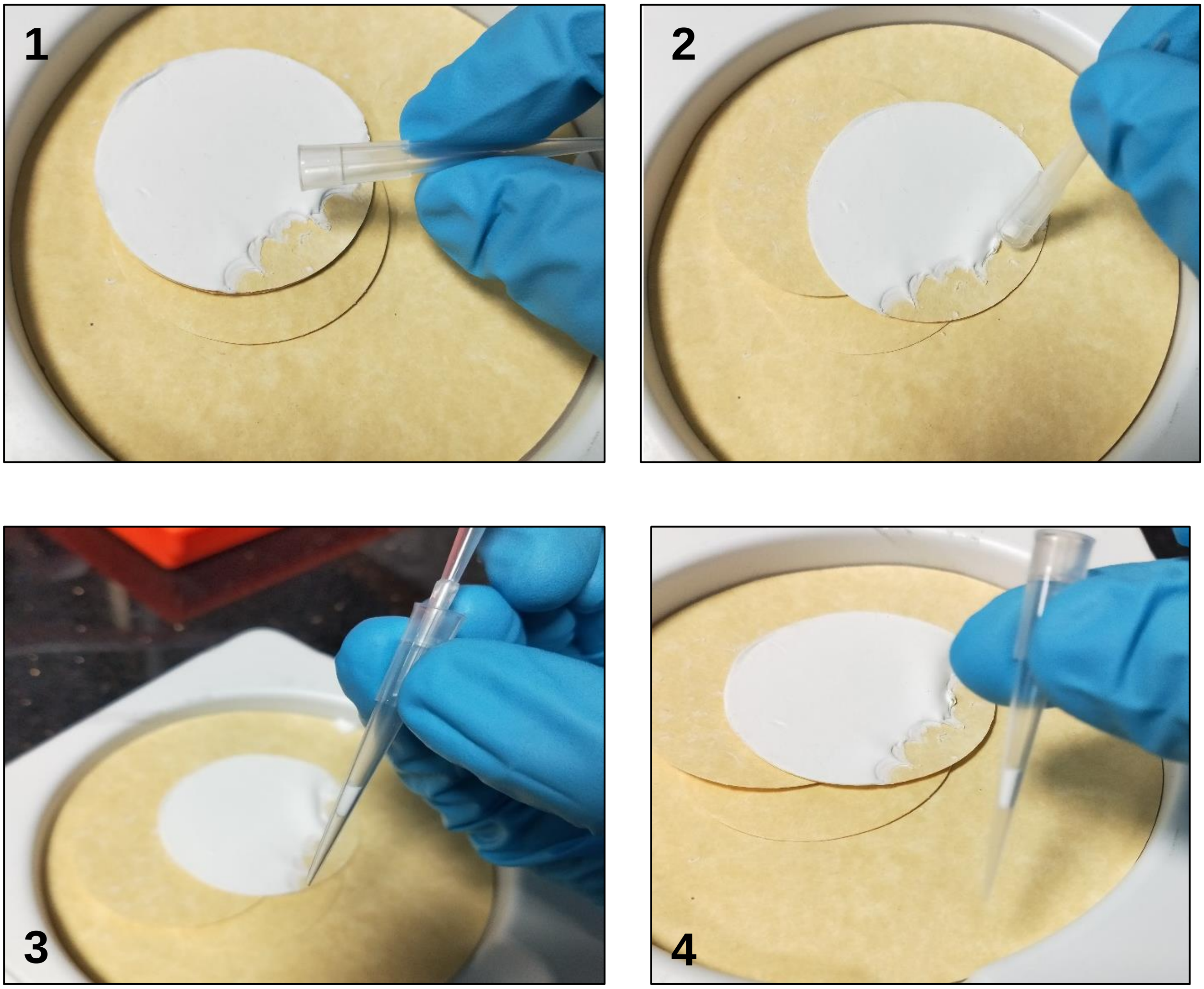

Figure S1 | Steps involved in the preparation of the C18 desalting tip.

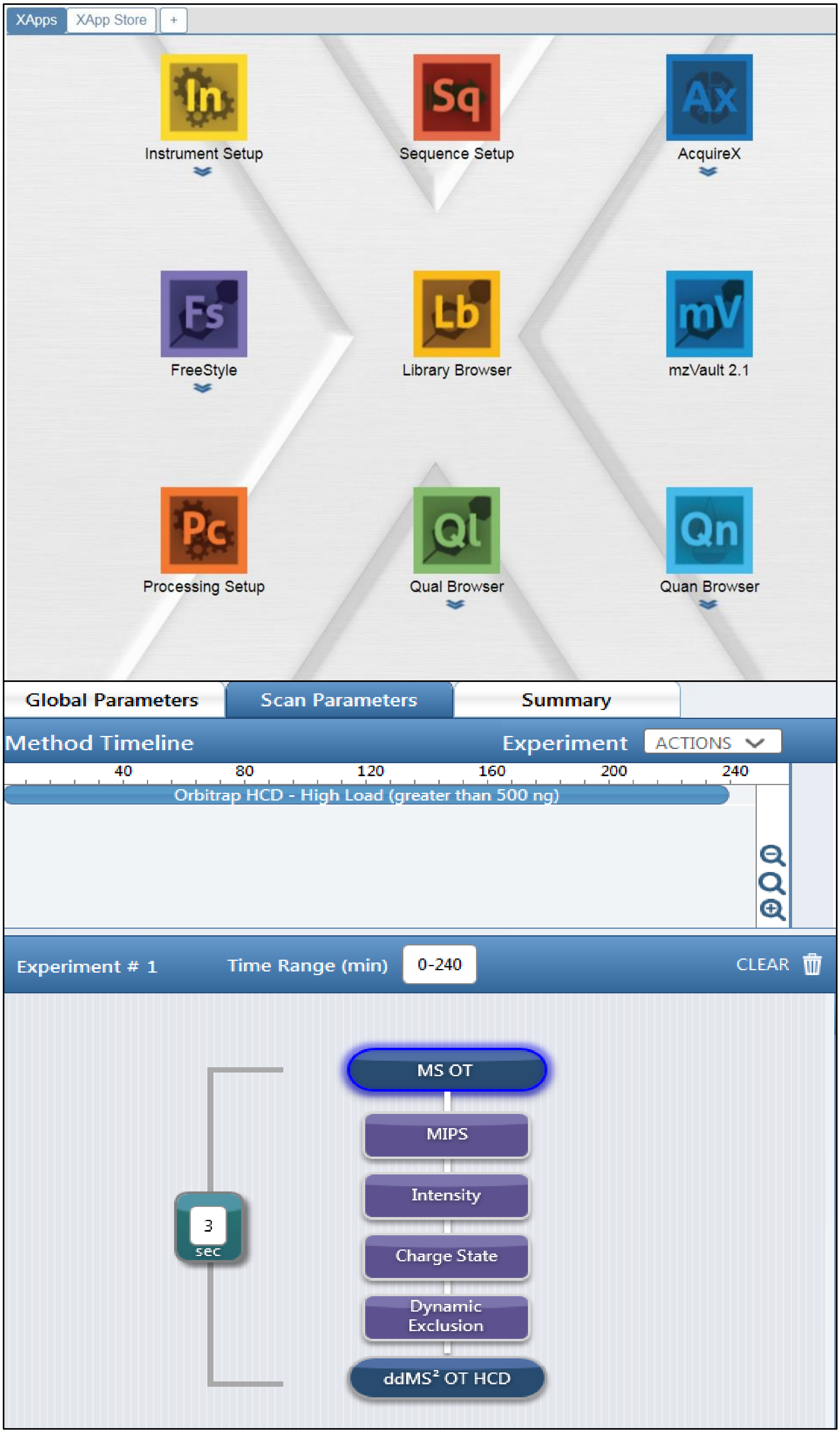

Figure S2 | Creating a data-dependent method using Thermo Xcalibur software.

```
Thermo EASY-LC method print for Fusion

 Sample pickup:

   Volume [µl]          : 3.00
   Flow [µl / min]      : 5.00

 Sample loading:

   Volume [µl]          : 15.00
   Flow [µl / min]      : (unspecified)
   Max. pressure [Bar]  : 750.00

 Gradient:

      Time [mm:ss]     Duration [mm:ss]     Flow [nl/min]     Mixture [%B]
             00:00                00:00            300.00             2.00
             05:00                05:00            300.00             5.00
             75:00                70:00            300.00            25.00
            105:00                30:00            300.00            45.00
            115:00                10:00            300.00            95.00
            120:00                05:00            300.00            95.00

 Pre-column equilibration:

   Volume [µl]          : 15.00
   Flow [µl / min]      : (unspecified)
   Max. pressure [Bar]  : 750.00

 Analytical column equilibration:

   Volume [µl]          : 10.00
   Flow [µl / min]      : (unspecified)
   Max. pressure [Bar]  : 750.00

 Autosampler wash:

   Flush volume [µl]    : 100.00
```

Method Summary

Method Settings

Application Mode: **Peptide**
Method Duration (min): **120**

Global Parameters

Ion Source

Use Ion Source Settings from Tune: **True**
FAIMS Mode: **Not Installed**

MS Global Settings

Default Charge State: **1**
Internal Mass Calibration: **User-defined Lock Mass**
Current Lock Mass: **Current**

Positive Ion

Positive Ion
m/z
445.12003

Negative Ion

Negative Ion
m/z

Experiment#1 [MS]

Start Time (min): **0**
End Time (min): **120**
Cycle Time (sec): **3**

Master Scan:

MS OT

Detector Type: **Orbitrap**
Orbitrap Resolution: **60000**
Mass Range: **Normal**
Use Quadrupole Isolation: **True**
Scan Range (m/z): **375-1700**
RF Lens (%): **60**
AGC Target: **4.0e5**
Maximum Injection Time (ms): **50**
Microscans: **1**
Data Type: **Profile**
Polarity: **Positive**
Source Fragmentation: **Disabled**
Scan Description:

Filters:

MIPS

Monoisotopic Peak Determination: **Peptide**

Charge State

Include charge state(s): **2-6**
Include undetermined charge states: **False**
Include charge states 25 and higher: **False**

Dynamic Exclusion

Exclude after n times: **1**
Exclusion duration (s): **40**
Mass Tolerance: **ppm**
Low: **10**
High: **10**
Exclude Isotopes: **True**
Perform dependent scan on single charge state per precursor only: **False**

Intensity

Filter Type: **Intensity Threshold**
Intensity Threshold: **5.0e3**

Data Dependent

Data Dependent Mode: **Cycle Time**
Time between Master Scans (sec): **3**

Scan Event Type 1:

Scan:

ddMS^2 OT HCD

Isolation Mode: **Quadrupole**
Isolation Window (m/z): **2**
Isolation Offset: **Off**
Activation Type: **HCD**
Collision Energy Mode: **Fixed**
HCD Collision Energy (%): **30**
Detector Type: **Orbitrap**
Scan Range Mode: **Auto: m/z Normal**
Orbitrap Resolution: **15000**
First Mass (m/z): **100**
AGC Target: **1.0e4**
Inject Ions for All Available Parallelizable Time: **True**
Maximum Injection Time (ms): **30**
Microscans: **1**
Data Type: **Centroid**
Use EASY-IC™: **False**
Scan Description:

Figure S3 | LC and MS parameters used for label-free quantitation.

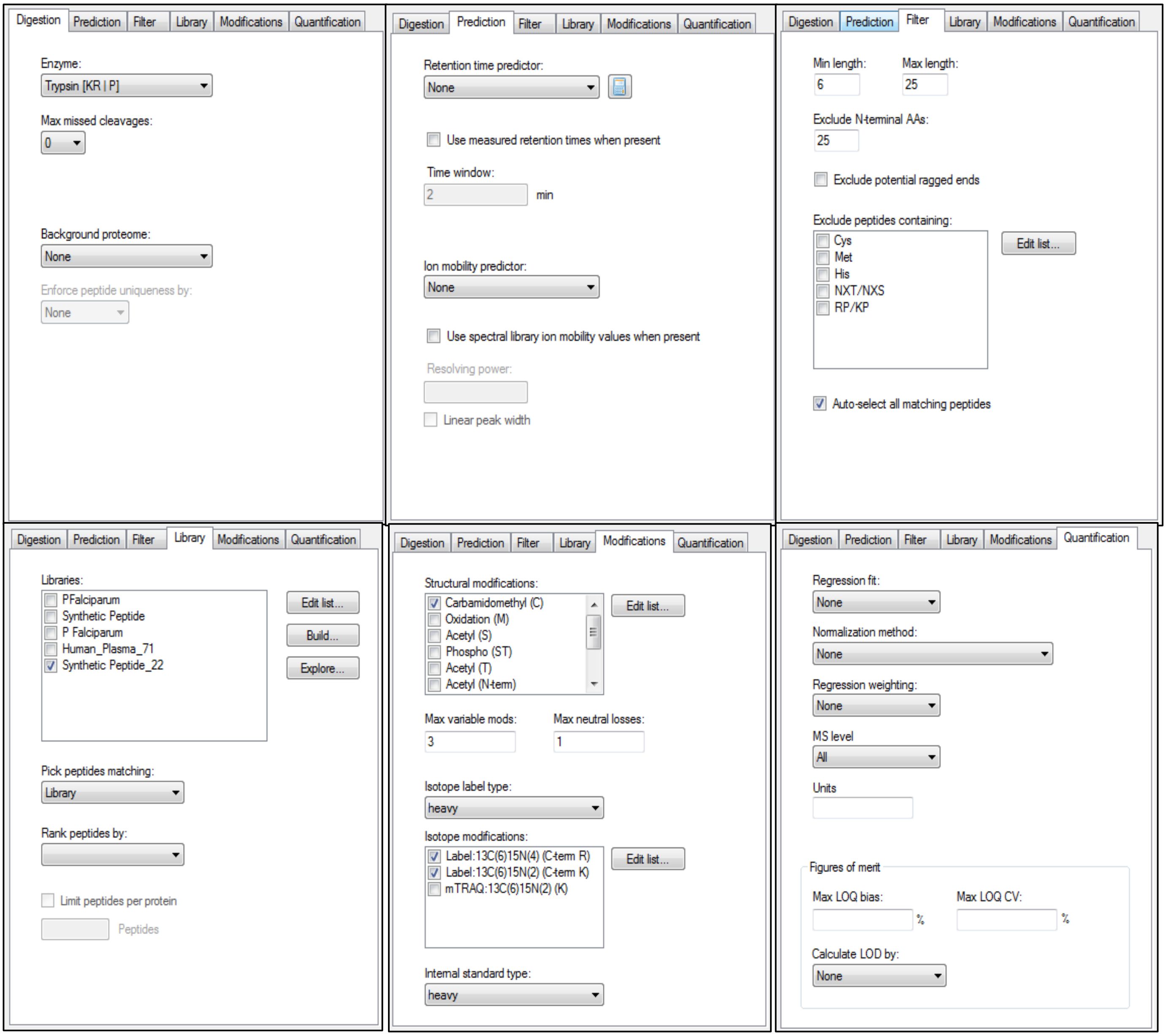

Figure S4 | Peptide settings for Multiple Reaction Monitoring and Parallel Reaction Monitoring using Skyline.

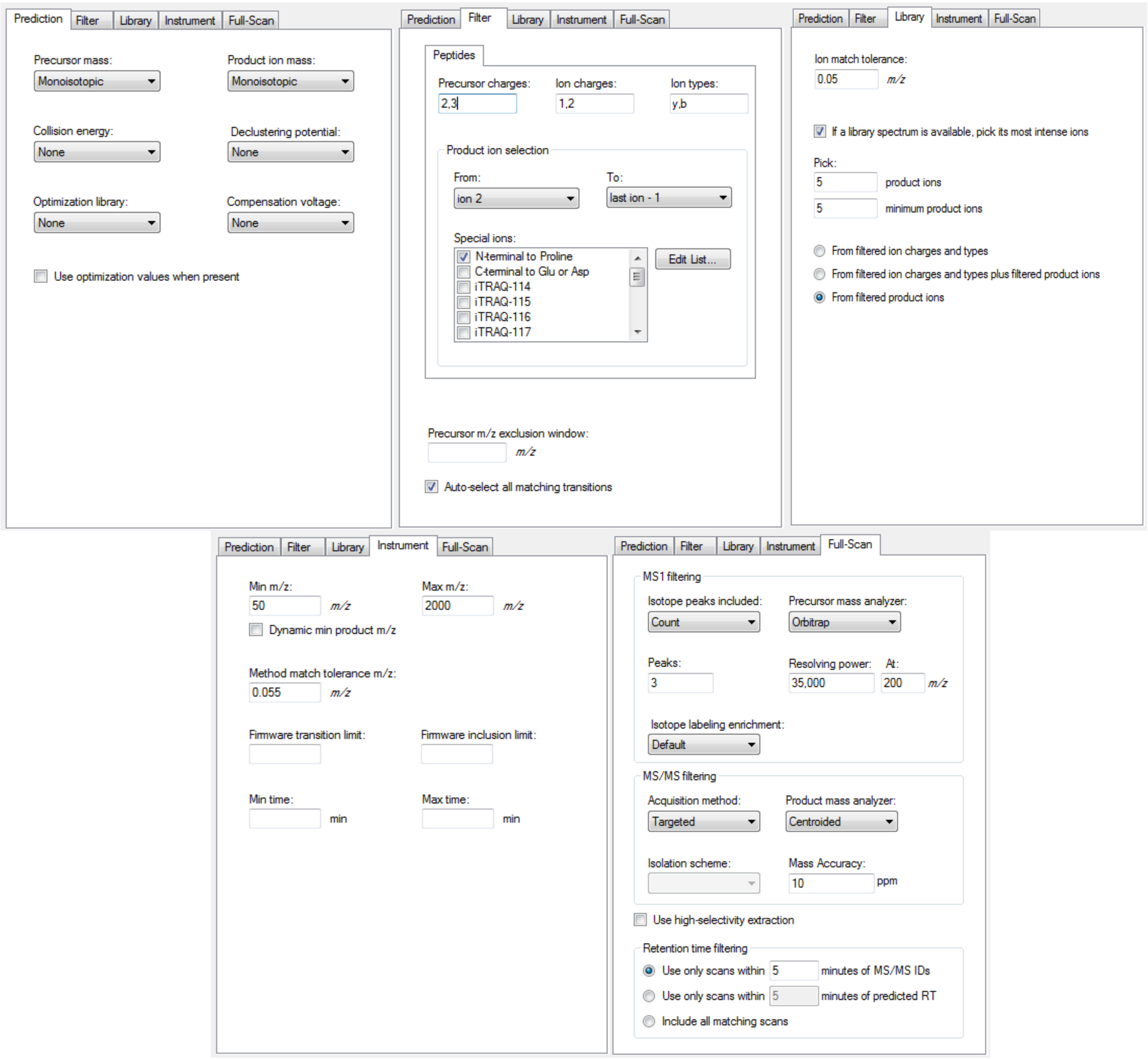

Figure S5 | Transition settings for Multiple Reaction Monitoring and Parallel Reaction Monitoring using Skyline

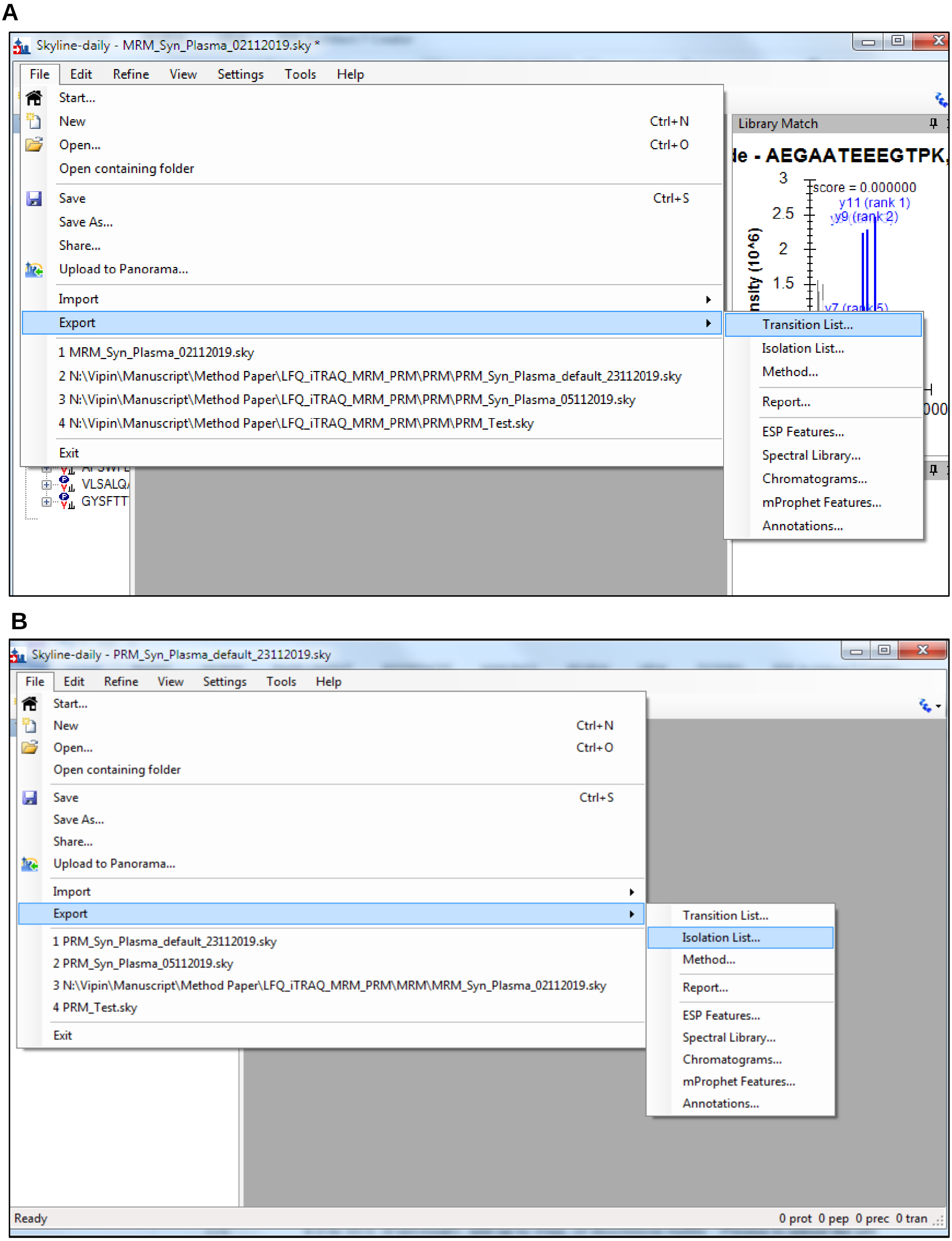

Figure S6 | (A,B) Procedure for exporting the list of peptides for Multiple Reaction Monitoring **(A)** and Parallel Reaction Monitoring experiment **(B)**.

Method Summary

Method Settings

Method Duration (min): **60**

Global Parameters

Ion Source

Ion Source Type: **NSI**
Spray Voltage: **Static**
Positive Ion (V): **2200**
Negative Ion (V): **600**

Positive Ion

Positive Ion	
Time (min)	Voltage (V)

Negative Ion

Negative Ion	
Time (min)	Voltage (V)

Sweep Gas (Arb): **0**
Ion Transfer Tube Temp (°C): **300**

Experiment 1

Start Time (min): **0**
End Time (min): **60**

Master Scan:

SRM

Use Cycle Time: **True**
Cycle Time (sec): **3**
Use Calibrated RF Lens: **True**
Q1 Resolution (FWHM): **0.7**
Q3 Resolution (FWHM): **0.7**
CID Gas (mTorr): **2**
Source Fragmentation (V): **0**
Chromatographic Peak Width (sec): **30**
Use Chromatographic Filter: **False**
Use Retention Time Reference: **False**
Display Retention Time: **False**
Use Quan Ion: **False**
Show Visualization: **False**

SRM Table

Compound	Start Time (min)	End Time (min)	Polarity	Precursor (m/z)	Product (m/z)	Collision Energy (V)	Min Dwell Time (ms)
AVLTIDEK (heavy)(+2)	0	60	Positive	448.762	399.197	18.4	15.274
AVLTIDEK (heavy)(+2)	0	60	Positive	448.762	512.281	18.4	15.274
AVLTIDEK (heavy)(+2)	0	60	Positive	448.762	613.328	18.4	15.274
AVLTIDEK (heavy)(+2)	0	60	Positive	448.762	726.412	18.4	15.274
LTWASHEK (heavy)(+2)	0	60	Positive	490.257	401.218	19.8	15.274
LTWASHEK (heavy)(+2)	0	60	Positive	490.257	421.229	19.8	15.274
LTWASHEK (heavy)(+2)	0	60	Positive	490.257	472.255	19.8	15.274
LTWASHEK (heavy)(+2)	0	60	Positive	490.257	508.261	19.8	15.274
LTWASHEK (heavy)(+2)	0	60	Positive	490.257	559.287	19.8	15.274
LTWASHEK (heavy)(+2)	0	60	Positive	490.257	579.298	19.8	15.274
LTWASHEK (heavy)(+2)	0	60	Positive	490.257	696.346	19.8	15.274
LTWASHEK (heavy)(+2)	0	60	Positive	490.257	765.377	19.8	15.274
FYQTVVHR (heavy)(+2)	0	60	Positive	530.284	375.218	21.2	15.274
FYQTVVHR (heavy)(+2)	0	60	Positive	530.284	421.255	21.2	15.274
FYQTVVHR (heavy)(+2)	0	60	Positive	530.284	439.198	21.2	15.274
FYQTVVHR (heavy)(+2)	0	60	Positive	530.284	520.323	21.2	15.274
FYQTVVHR (heavy)(+2)	0	60	Positive	530.284	540.245	21.2	15.274
FYQTVVHR (heavy)(+2)	0	60	Positive	530.284	621.371	21.2	15.274
FYQTVVHR (heavy)(+2)	0	60	Positive	530.284	639.314	21.2	15.274
FYQTVVHR (heavy)(+2)	0	60	Positive	530.284	738.382	21.2	15.274
FYQTVVHR (heavy)(+2)	0	60	Positive	530.284	749.429	21.2	15.274
SADTLWGIQK (heavy)(+2)	0	60	Positive	563.802	396.27	22.3	15.274
SADTLWGIQK (heavy)(+2)	0	60	Positive	563.802	453.291	22.3	15.274
SADTLWGIQK (heavy)(+2)	0	60	Positive	563.802	639.37	22.3	15.274
SADTLWGIQK (heavy)(+2)	0	60	Positive	563.802	752.454	22.3	15.274
SADTLWGIQK (heavy)(+2)	0	60	Positive	563.802	853.502	22.3	15.274

Figure S7 | Method Parameters for Multiple Reaction Monitoring experiment on TSQ Altis.

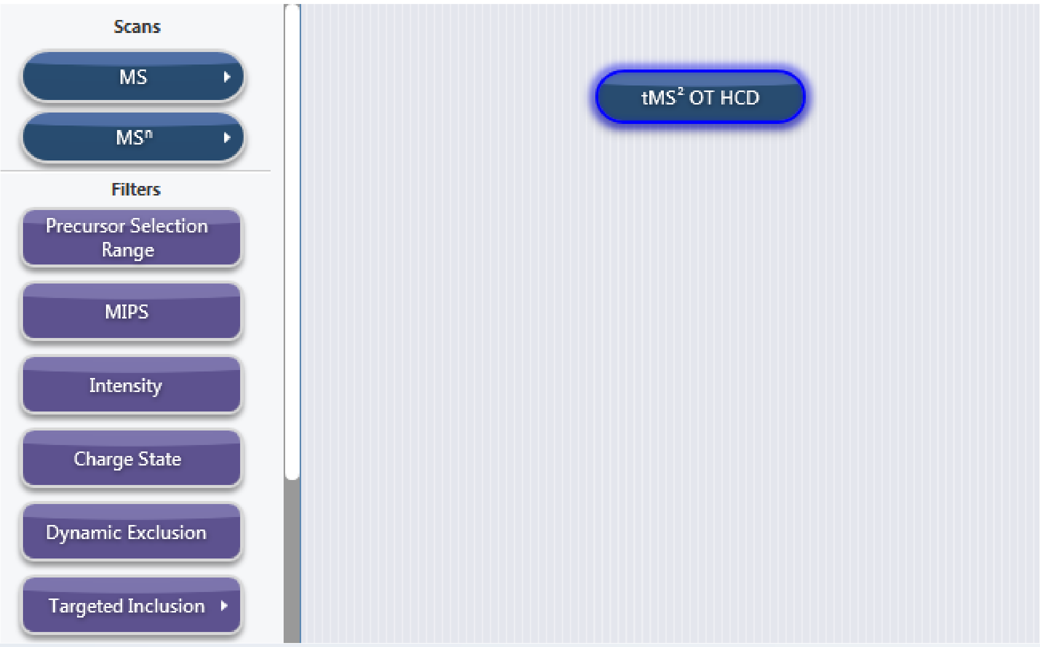

Method Summary

Method Settings

Application Mode: **Peptide**
Method Duration (min): **60**

Global Parameters

Ion Source

Use Ion Source Settings from Tune: **True**
FAIMS Mode: **Not Installed**

MS Global Settings

Default Charge State: **2**
Internal Mass Calibration: **Off**

Experiment#1 [tMSn]

Start Time (min): **0**
End Time (min): **60**

Master Scan:

tMS^2 OT HCD

MS^n Level (n): **2**
Multiplex Ions: **False**
Isolation Mode: **Quadrupole**
Isolation Window (m/z): **1**
Activation Type: **HCD**
HCD Collision Energy (%): **30**
Stepped Collision Energy: **False**
Detector Type: **Orbitrap**
Orbitrap Resolution: **60000**
Mass Range: **Normal**
Scan Range (m/z): **350-1700**
RF Lens (%): **60**
AGC Target: **1.0e5**
Inject Ions for All Available Parallelizable Time: **False**
Maximum Injection Time (ms): **118**
Microscans: **1**
Data Type: **Centroid**
Polarity: **Positive**
Source Fragmentation: **Disabled**
Use EASY-IC™: **False**
Loop Control: **All**
Scan Description:
Include Start/End Times: **False**

Mass List Table

Mass List Table

Compound	Formula	m/z	z
FYQTVVHR (heavy)		530.2841	2
AEQEHWEEF EEK (heavy)		799.8461	2
VFPYDNTLP K (heavy)		601.3208	2
GVSGLVPSS NSLQEITLR (heavy)		934.0116	2
DNIFITNVLD QWIK (heavy)		863.9663	2
LTWASHEK (heavy)		490.258	2
SLYASSPGG VYATR (heavy)		719.8637	2
SADTLWGIQ K (heavy)		563.8028	2
IESLDYLSLFK (heavy)		668.3679	2
SDGAPASDS KPGSSEAAP SSK (heavy)		970.9498	2
AEGAATEEE GTPK (heavy)		649.3035	2
ESEPQAAAE PAEAK (heavy)		718.3432	2
ETPAATEAP SSTPK (heavy)		697.8481	2
EEKPAVTAA PK (heavy)		574.8237	2
ITIPVQTFSN LQIR (heavy)		820.4739	2

Figure S8 | Method parameters for the Parallel Reaction Monitoring experiment on Orbitrap Fusion.

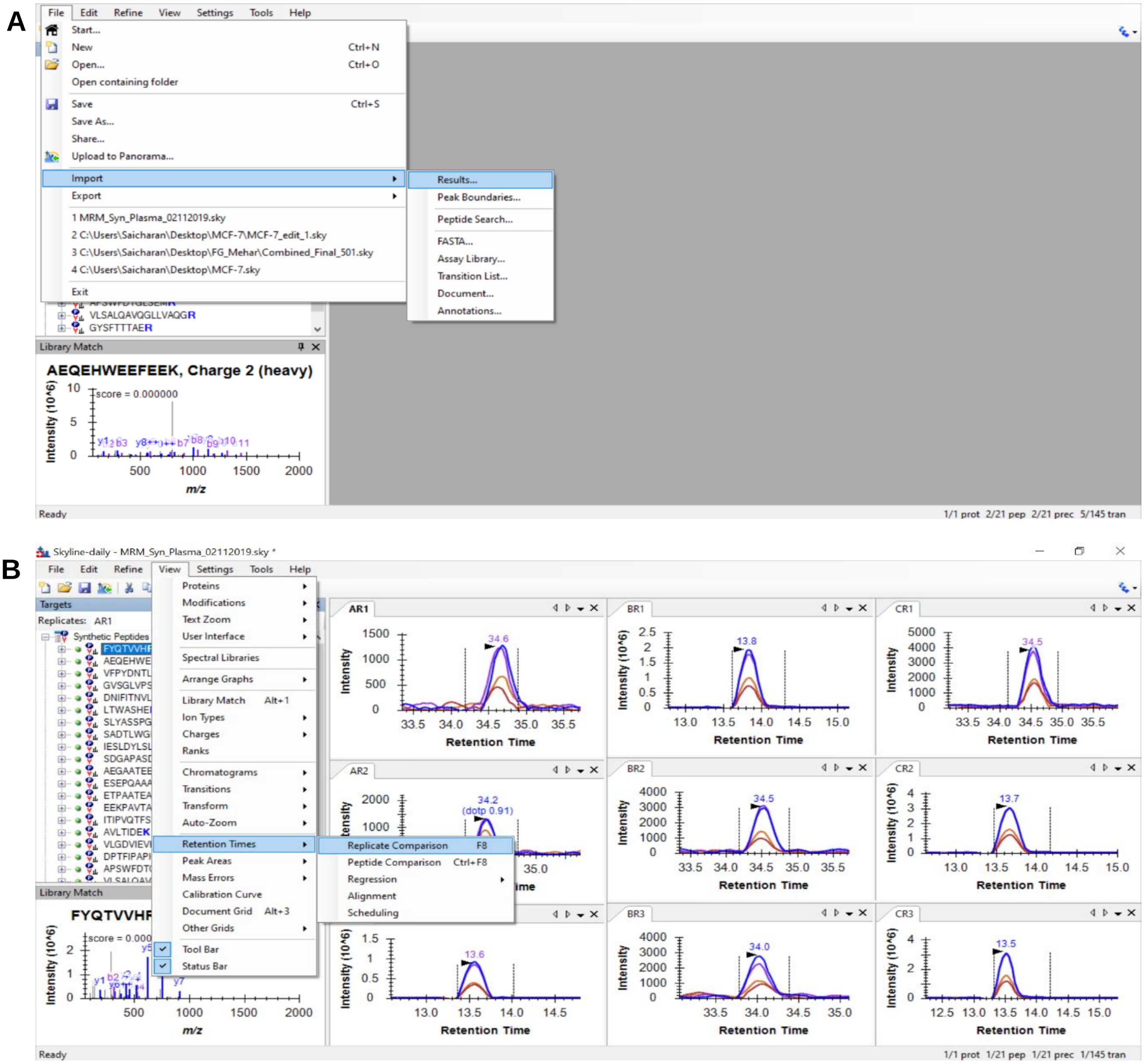

Figure S9 | Data analysis using Skyline. (A) Importing the result files into the Skyline document. **(B)** Steps to view the retention time of peptides.

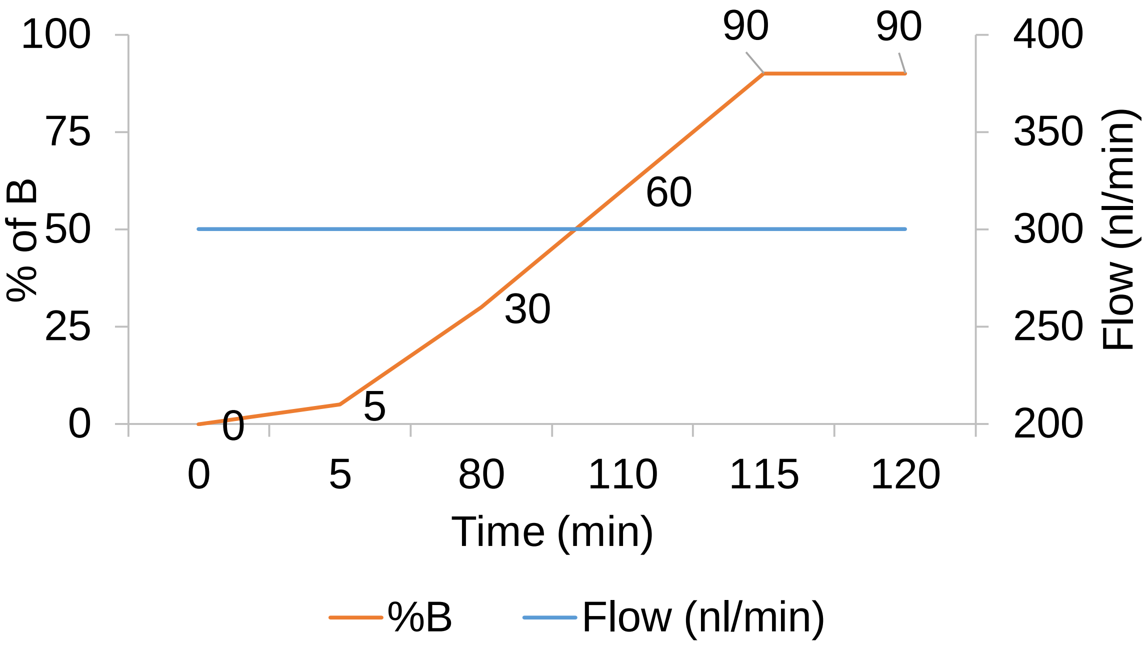

	AR1	AR2	AR3	BR1	BR2	BR3	CR1	CR2	CR3
AR1	1.000	0.999	0.999	0.993	0.991	0.992	1.000	0.999	0.999
AR2	0.999	1.000	1.000	0.994	0.990	0.993	0.999	0.999	1.000
AR3	0.999	1.000	1.000	0.994	0.990	0.993	0.999	0.999	1.000
BR1	0.993	0.994	0.994	1.000	0.997	0.999	0.991	0.991	0.993
BR2	0.991	0.990	0.990	0.997	1.000	0.999	0.990	0.990	0.989
BR3	0.992	0.993	0.993	0.999	0.999	1.000	0.991	0.991	0.992
CR1	1.000	0.999	0.999	0.991	0.990	0.991	1.000	1.000	0.999
CR2	0.999	0.999	0.999	0.991	0.990	0.991	1.000	1.000	0.999
CR3	0.999	1.000	1.000	0.993	0.989	0.992	0.999	0.999	1.000

Figure S10 | (A) The gradient used for a 2-hour separation through liquid chromatography. **(B)** Correlation analysis of the technical replicates (R1, R2, R3) of three different biological pools of plasma samples (Sample A, B and C).

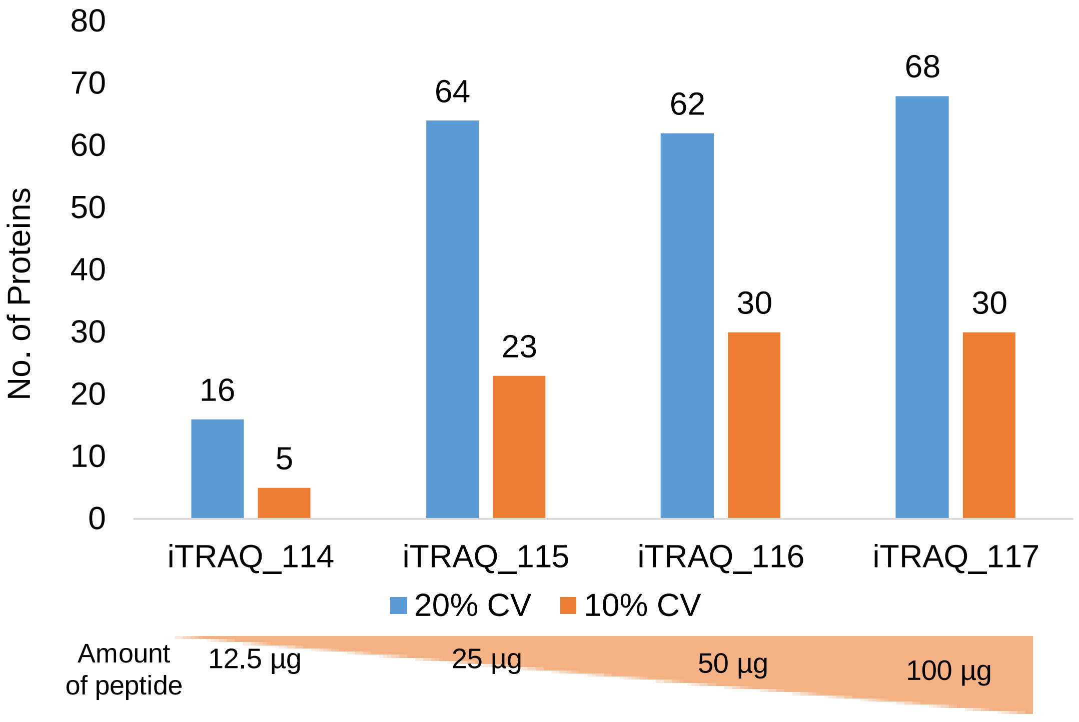

Figure S11 | The total no. proteins which has a coefficient of variation of less than 20% and 10% in each of the iTRAQ 4-plex labels- 114, 115, 116, and 117 after applying 1% FDR. The number of labeled peptides varied across the iTRAQ reagents.

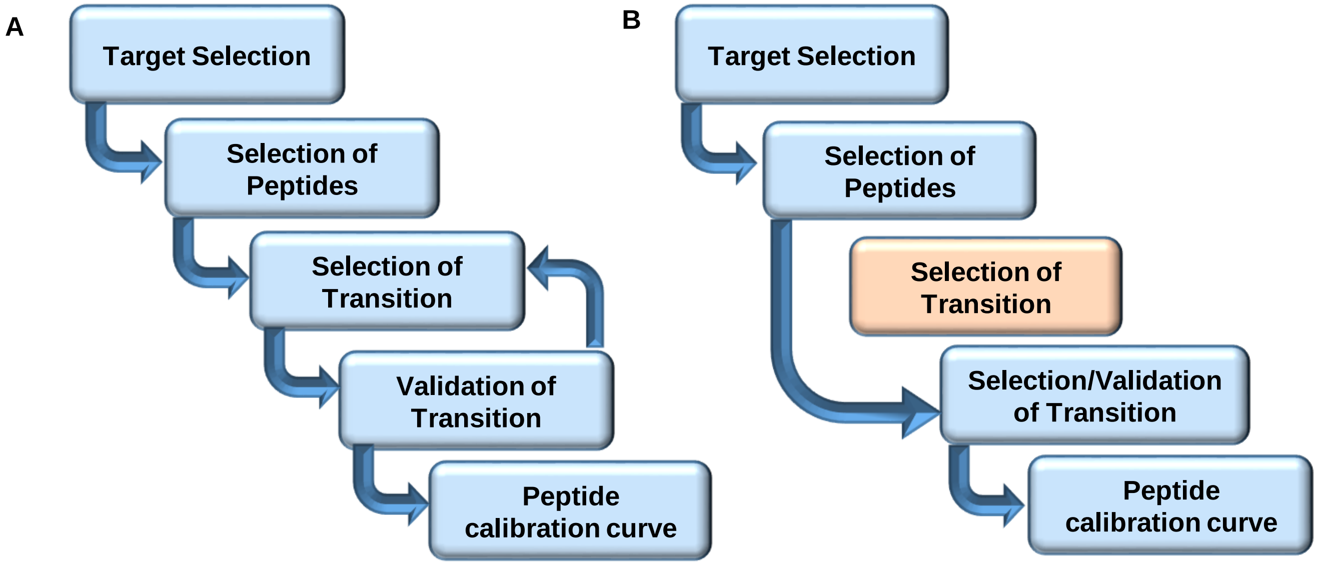

Figure S12 | Targeted proteomics using MS-based approaches. (**A, B**) Workflow for Multiple Reaction Monitoring (MRM), this approach consider the transitions of the peptide (**A**) and Parallel Reaction Monitoring (PRM), this approach consider only selection of peptides **(B)**. It is independent of transitions.

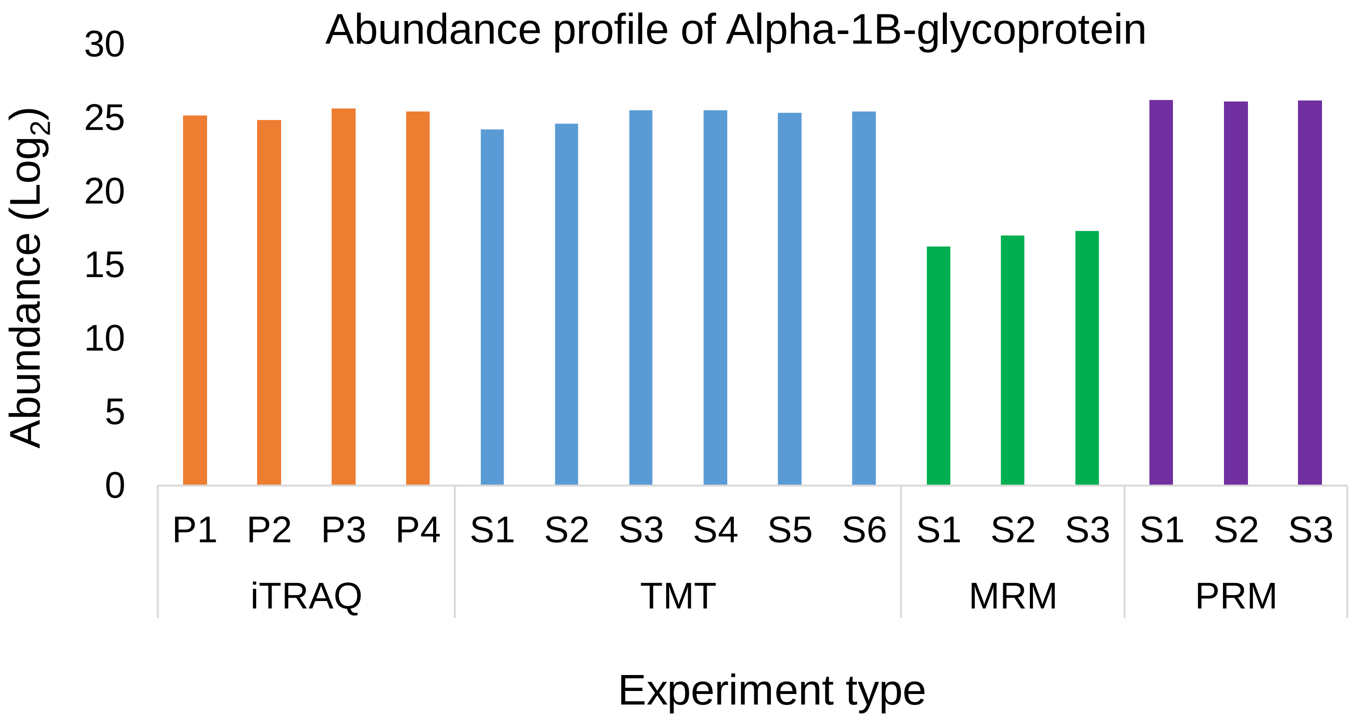

Figure S13 | The abundance profile of alpha-1B-glycoprotein in the pooled samples (iTRAQ experiment) and individual samples (TMT, MRM, and PRM experiments) using different proteomic techniques. The y-axis represents the abundance in Log_2, and the x-axis represents samples used in iTRAQ, TMT, MRM, and PRM experiments. The protein abundance of alpha-1B-glycoprotein is the average abundance of three peptides (R.SGLSTGWTQLSK.L, R.CEGPIPDVTFELLR.E, and R.TPGAAANLELIFVGPQHAGNYR.C) in case of MRM and PRM experiments. P: Pooled sample, S: individual sample.

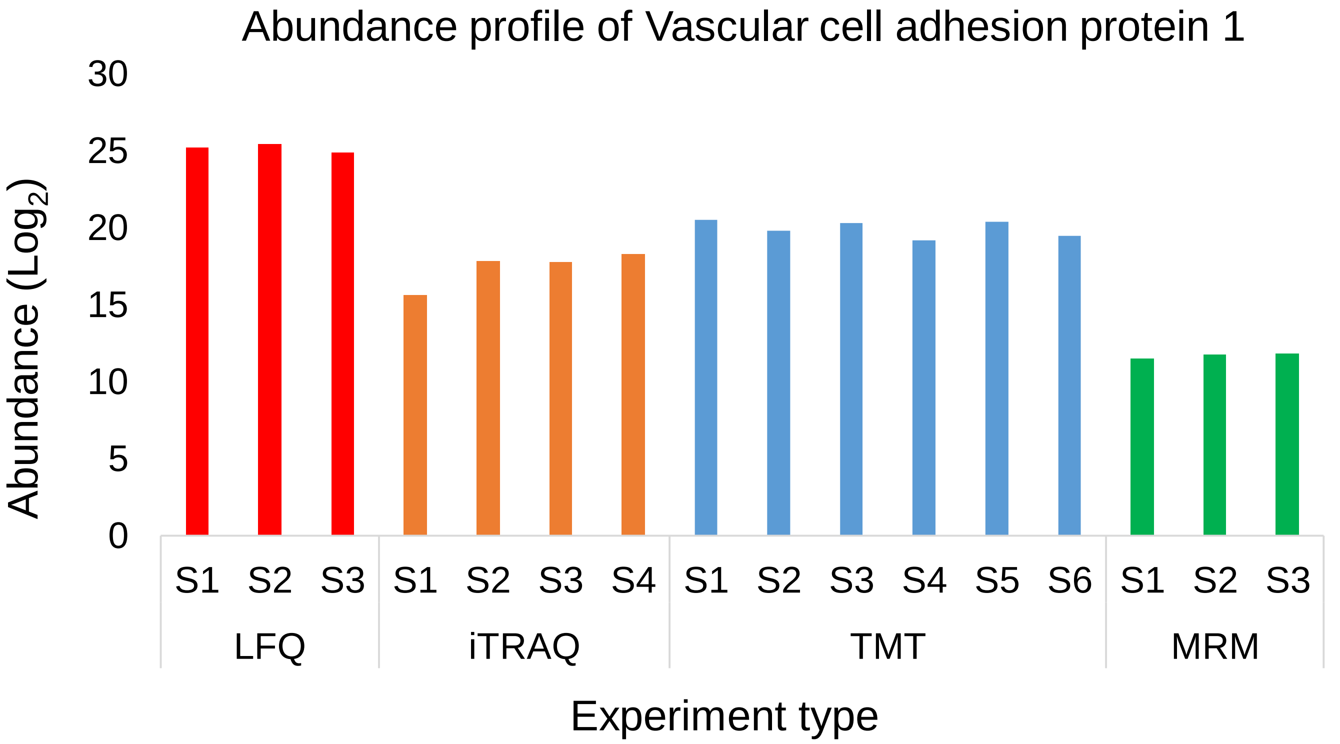

Figure S14 | The abundance profile of vascular cell adhesion protein 1 in label-free quantitation (LFQ), iTRAQ-4plex, TMT-6plex, and MRM experiments. The y-axis represents the abundance in Log_2, and the x-axis represents samples used in LFQ, iTRAQ-4plex, and TMT-6plex experiments. S: Plasma sample
